# Selective Structural Derivatization of Flavonoid Acetamides Significantly Impacts Their Bioavailability and Antioxidant Properties

**DOI:** 10.3390/molecules27238133

**Published:** 2022-11-22

**Authors:** Daniel Kasungi Isika, Omowunmi A. Sadik

**Affiliations:** Department of Chemistry and Environmental Science, BioSensor Materials for Advanced Research & Technology (BioSMART Center), New Jersey Institute of Technology, 161 Warren Street, University Heights, Newark, NJ 07102, USA

**Keywords:** flavonoid, acetamide, bioavailability, antioxidant, structure-activity relationship, HPLC-MS, ADMET, quercetin, apigenin, luteolin

## Abstract

Flavonoids show abundant favorable physicochemical and drug related properties, leading to substantial biological applications which are limited by undesirable properties such as poor solubility, high polarity, low bioavailability, and enzymatic degradations. Chemical modification with bioisosteres can be used to address some of these challenges. We report the synthesis and characterization of partial flavonoid acetamide derivatives from quercetin, apigenin and luteolin and the evaluation of their structure-activity relationships based on antioxidant, bioavailability, drug likeness, and toxicity properties. The sequential synthesis was achieved with 76.67–87.23% yield; the structures of the compounds were confirmed using ^1^H & ^13^C NMR characterizations. The purity of each compound was determined by HPLC while the molecular weights were determined by mass spectrometry. The % bioavailability was determined using the dialysis tubing procedure and the values were in the range 15.97–38.12%. The antioxidant activity was determined by the 2,2-diphenyl-1-picrylhydrazyl (DPPH) assay and expressed as the IC_50_ values which were in the range 31.52–198.41 µM. The drug likeness and the toxicity properties of compounds 4, 5, 7, 11 and 15 were predicted using computational tools and showed satisfactory results. A structure-activity relationship evaluation reveals that hydroxyl and methylene groups attached on the 2-phenylchromen-4-one structure of the flavonoid play a colossal role in the overall antioxidant and bioavailability properties. The improved bioavailability and excellent drug relevance and toxicity properties present flavonoid acetamide derivatives as prospective drug candidates for further evaluations.

## 1. Introduction

Flavonoids are a class of naturally occurring hydroxylated polyphenolic plant compounds [1,2]. They exist in numerous classes such as flavones, flavanones, flavonols and isoflavones [1,3]. Flavonoids are chemically distinguishable by their 15-carbon skeleton in which two phenyl rings are interlinked through a three-carbon chain in the form of a heterocyclic ring [3,4]. Different classes of flavonoids exhibit distinctive physicochemical properties and thus distinct biological activities. Flavonoids are freely available in vegetables such as onions, spinach and kales, as well as in fruits such as tomatoes, cherries, blueberries and grapes [2]. The flavonoid content in vegetables and fruits greatly varies depending on the geological location, stage of growth, and class of plants, among others. The different classes of flavonoids form an integral part of the human diet given their ample biological applications [5,6,7].

Flavonoids have extensive biological applications. These include antibacterial [8], anticancer [9,10] antioxidant [11], anti-inflammatory [12,13], and enzyme inhibition [14,15,16,17]. The extent of the biological activity of these flavonoids depends on the physicochemical properties of the compounds, which vary depending on the class of the flavonoids. The extent of the biological functions of the flavonoids are constrained by some unsought physicochemical; hence, biological properties. These limitations include poor solubility, enzymatic degradation, low bioavailability, and low antioxidant activity [4,18,19,20,21].

Most undesired flavonoid biological activities may be linked to the physicochemical properties of the flavonoids themselves, while some undesired properties may arise from the metabolites, fragments, and derivatives of the flavonoids. Enzymatic and chemical degradation and reactions on the flavonoids may yield derivatives which not only reduce the expected biological activity but also yield undesirable toxins. The presence of reactive groups in the flavonoids makes it possible to interact with certain groups thus converting these flavonoids into different classes of compounds. Enzymes may degrade the flavonoids into metabolites, such as quinones, or convert them into flavonoid fragments and derivatives with unsolicited properties through glycosylation, hydroxylation, and methylation processes [22,23].

Flavonoids can be modified to enhance the desired properties and manage the undesired properties. This modification can be achieved through both partial and global chemical modifications. The chemical modifications of flavonoids have been done principally to manage the enzymatic degradation, bioavailability, solubility, and polarity, as well as to introduce functional groups with preferred properties. The desired functional groups in flavonoids can be achieved through the modifications of the flavonoid’s carbonyl group, hydroxyl group, phenyl, and pyran rings [4,24].

Bioisosteres have been largely used in the modification of the flavonoid hydroxyl and carbonyl groups. Flavonoids have been modified into different classes of compounds through metal coordination [25,26,27], functionalization of the phenyl rings [4], O-alkylation [28,29], O-acylation [30,31], and the modification of the hydroxyl groups [4]. Owing to the multiple rings and hydroxyl groups in the flavonoids, bioisosteric modification can be achieved through either global or partial modifications. Thus, structure-activity relationships studies are essential in the determination of the effects of these modifications on select flavonoid properties [4].

Most of the flavonoid biological properties and applications are anchored on the bioavailability and antioxidant properties. These two properties determine the extent of the effectiveness of the flavonoids in biological applications. The bioavailability of the flavonoids is a measure of the amount of the flavonoids that will reach their target site, the circulation system, and manage the intended biological activity. Like drug molecules, when flavonoids are orally introduced into the body, they are exposed to many enzymatic and chemical changes before entering the circulation system. The enzymatic degradation of flavonoids into metabolites and the chemical modification of the flavonoids into other flavonoid derivatives will certainly alter the physicochemical, and hence the biological properties, of the flavonoids [32,33]. It is critical to investigate the effects of such changes on the bioavailability through structure activity studies. The antioxidant capacity of flavonoids contributes to various biological properties of the flavonoids, such as anticancer properties. Electron delocalization and radical formations are key pathways through which flavonoid antioxidant activity is achieved [34]. The antioxidant capacity of the flavonoids is also a factor of hydrogen bonding, electronic interactions, and molecular forces. These forces change upon the disruption of the electronic structure of the flavonoids, and it is imperative that the overall antioxidant activity of flavonoid derivatives be determined [35]. This can be achieved through structure-activity relationship studies on the antioxidant activity of flavonoids and flavonoid derivatives.

Flavonoids have been studied for their ability to scavenge free radicals. The hydroxyl groups in the flavonoids enable them to scavenge free radicals through the reduction-oxidation mechanisms. The electron delocalization across the flavonoid polyphenolic and heterocyclic rings, as well as the hydroxyl groups, allow the flavonoids to act as antioxidant agents. Flavonoids are chemically favored as single electron donors. They can stabilize peroxyl, superoxide, and hydroxyl radicals predominantly through the donation of a hydrogen atom. Both kinetic and thermodynamic properties play a key role in the extent to which flavonoids react with and stabilize free radicals [36,37,38].

To address the predominant challenges in flavonoids, we designed and synthesized a class of flavonoid derivatives called the flavonoid acetamide derivatives. In these derivatives, the main targets are the hydroxyl groups at positions C-5, C3′ and C4′; which are the main ones that determine the electron flow, and hence the antioxidant properties. The acetamide derivative comprises sp^3^ hybridized methylene groups which are essential in the regulation of the flexibility, polarity, and lipophilicity of the flavonoids. The acetamide group further has the amide group which facilitates hydrogen bonding and molecular interactions through the delocalized electrons. The polarity, electron delocalization, hydrogen bond formations, and lipophilicity are all crucial factors in the determination of the overall bioavailability and antioxidant capacity, thus regulating the biological applications of flavonoids. We previously reported a group of compounds in which all the hydroxyl groups in the flavonoid were converted into the acetamide group; such compounds are generally referred to as global flavonoid acetamide derivatives [39,40].

In this study, selected hydroxyl groups in the flavonoid were converted into acetamide groups; thus, these derivatives are generally referred to as partial flavonoid acetamide derivatives. Quantitative control was used, which relies on the use of exact reagents, especially when temperature and steric effects influence the reaction rates. The physicochemical properties of the flavonoids—hence their biological properties and applications—are highly dependent on both the presence and position of different functional groups in the flavonoids. It is therefore critical that the structure-activity relationships of the flavonoids be determined to ascertain the effect of the presence and locations of different functional groups on some selected properties. In this study, we evaluated the structure-activity relationships of the partially modified flavonoid acetamide derivatives on the bioavailability and antioxidant activity.

In this study, we synthesized and characterized partially derived flavonoid acetamide derivatives. The flavonoid acetamide derivatives in this study were 2,2′-((4-(3,7-bis(2-amino-2-oxoethoxy)-5-hydroxy-4-oxo-4H-chromen-2-yl)-1,2-phenylene)bis(oxy))diacetamide or quercetin tetra acetamide (**4**), 2-(4-(7-(2-amino-2-oxoethoxy)-5-hydroxy-4-oxo-4H-chromen-2-yl)phenoxy)acetamide or apigenin di acetamide (**11**), 2,2′-((4-(7-(2-amino-2-oxoethoxy)-5-hydroxy-4-oxo-4H-chromen-2-yl)-1,2-phenylene)bis(oxy))diacetamide or luteolin tri acetamide (**15**), ethyl 2-((3,7-bis(2-amino-2-oxoethoxy)-2-(3,4-bis(2-amino-2-oxoethoxy)phenyl)-4-oxo-4H-chromen-5-yl)oxy)acetate (**5**), and 2,2′,2″-((2-(2,3-dihydrobenzo[b][1,4]dioxin-6-yl)-4-oxo-4H-chromene-3,5,7-triyl)tris(oxy))triacetamide (**7**). The structures of these compounds were confirmed through ^1^H & ^13^C NMR analyses. The purity of these compounds was analyzed using high-performance liquid chromatography (HPLC) characterization. The masses of the compounds were found using mass spectrometry characterization. The bioavailability and antioxidant activity were determined through in vitro dialysis tubing procedure and the 2,2-diphenyl-1-picrylhydrazyl (DPPH) radical scavenging assay, respectively. The drug likeness and relevant properties, as well as the absorption, distribution, metabolism, excretion, and toxicity (ADMET) properties were determined computationally.

## 2. Materials and Methods

High purity grade lithium hydroxide monohydrate, esterase, acetone, ethyl chloroacetate, ethyl acetate, dimethylformamide, sodium cholate, anhydrous potassium carbonate, hydrochloric acid, formic acid, acetic acid, amylase, thionyl chloride, ammonia solution, hexane, sodium bicarbonate, phosphate-buffered saline (PBS) buffer, sodium chloride, sodium sulfate, potassium iodide, dichloromethane, dialysis tubing, porcine pancreatin, silica gel, dimethyl sulfoxide, acetonitrile, porcine pepsin, 2-chloroacetamide, and 1,2-dibromoethane were purchased from Millipore Sigma (Burlington, MA, USA). The high purity grade apigenin, quercetin and luteolin were obtained from Indofine chemical company (Hillsborough, NJ, USA).

### 2.1. Selective Synthesis of Flavonoid Acetamide Derivatives

**Scheme 1:**
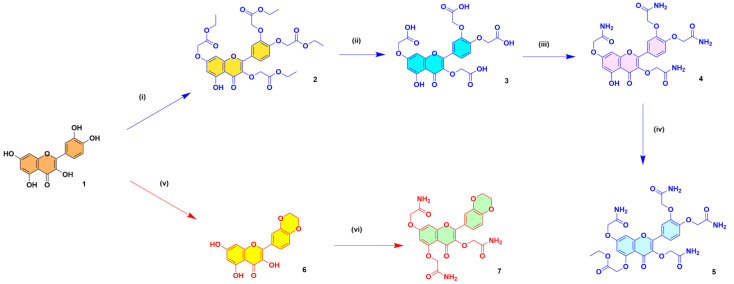
Steps in the selective synthesis of 2,2′-((4-(3,7-bis(2-amino-2-oxoethoxy)-5-hydroxy-4-oxo-4H-chromen-2-yl)-1,2-phenylene)bis(oxy))diacetamide or quercetin tetra acetamide (**4**), ethyl 2-((3,7-bis(2-amino-2-oxoethoxy)-2-(3,4-bis(2-amino-2-oxoethoxy)phenyl)-4-oxo-4H-chromen-5-yl)oxy)acetate (**5**) and 2,2′,2″-((2-(2,3-dihydrobenzo[b][1,4]dioxin-6-yl)-4-oxo-4H-chromene-3,5,7-triyl)tris(oxy))triacetamide (**7**) derivatives.

The flavonoid acetamide derivatives were synthesized using our previously reported optimized procedure [39,40]. The structures of the compounds were confirmed using ^1^H & ^13^C NMR analyses and electrospray ionization mass spectrometry (ESI-MS) characterizations. The purity of each compound was determined using reverse phase high performance liquid chromatography (RP-HPLC). The detailed step-by-step procedure used in the synthesis of the final flavonoid acetamide derivatives, as well as the prevalent intermediates, is discussed below. The reaction conditions, as well as the structures of the intermediates and the final products in the synthesis of compounds **4**, **5** and **7**, are shown in Scheme 1.

Reagents and reaction conditions. Synthesis of compound **4**: (i) Ethyl chloroacetate (3.75 eq.), K_2_CO_3_ (4 eq.), DMF, Rt. (ii) LiOH.H_2_O (4 eq.), THF/H_2_O, Rt. (iii) SOCl_2_ (4 eq.), reflux; NH_4_OH (4 eq.), 0 °C—Rt. Synthesis of compound **5**: (iv) Ethyl chloroacetate (1.5 eq.), K_2_CO_3_ (1.5 eq.), DMF, Rt. Synthesis of compound **7**: (v) 1,2-dibromoethane (2.15 eq.), K_2_CO_3_ (0.575 eq.), 100 °C. (vi) K_2_CO_3_ (3 eq.), 2-chloroacetamide (3.5 eq.), DMF, KI, Rt.

**Scheme 2:**
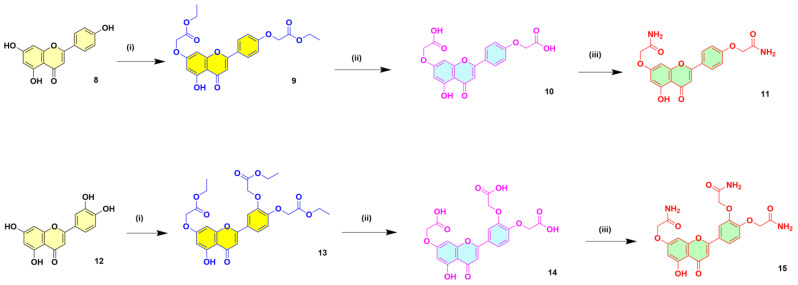
Steps in the selective synthesis of 2-(4-(7-(2-amino-2-oxoethoxy)-5-hydroxy-4-oxo-4H-chromen-2-yl)phenoxy) acetamide or apigenin di acetamide (**11**) and 2,2′-((4-(7-(2-amino-2-oxoethoxy)-5-hydroxy-4-oxo-4H-chromen-2-yl)-1,2-phenylene) bis(oxy))diacetamide or luteolin tri acetamide (**15**) derivatives.

The synthesis of compounds **11** and **15** was equally achieved following our previously reported optimized procedure [39,40]. The experimental steps and the reaction conditions are all explained in the procedure section. The optimized reaction conditions, as well as the structures of the intermediates and the final products in the synthesis of compounds **11** and **15**, are shown in Scheme 2.

Reagents and reaction conditions. Synthesis of compound **11**: (i) Ethyl chloroacetate (2 eq.), K_2_CO_3_ (2 eq.), DMF, Rt. (ii) LiOH.H_2_O (2 eq.), THF/H_2_O, Rt. (iii) SOCl_2_ (2 eq.), reflux, NH_4_OH (2 eq.), 0 °C—Rt. Synthesis of compound **15**: (i) Ethyl chloroacetate (3 eq.), K_2_CO_3_ (3 eq.), DMF, Rt. (ii) LiOH.H_2_O (3 eq.), THF/H_2_O, Rt. (iii) SOCl_2_ (3 eq.), reflux, NH_4_OH (3 eq.), 0 °C—Rt.

Synthesis of diethyl 2,2′-((4-(3,7-bis(2-ethoxy-2-oxoethoxy)-5-hydroxy-4-oxo-4H-chromen-2-yl)-1,2-phenylene)bis(oxy))diacetate (**2**); ethyl 2-(4-(7-(2-ethoxy-2-oxoethoxy)-5-hydroxy-4-oxo-4H-chromen-2-yl)phenoxy)acetate (**9**) and diethyl 2,2′-((4-(7-(2-ethoxy-2-oxoethoxy)-5-hydroxy-4-oxo-4H-chromen-2-yl)-1,2-phenylene)bis(oxy))diacetate (**13**).

The synthesis of compounds **2**, **9** and **13** was achieved following our previously reported procedure [39,40]. In an oven dried flask, quercetin, compound **1**, (500 mg, 1.654 mmol, 1 eq.) was dissolved in dry dimethylformamide (DMF) forming a yellow solution. Into this solution was added anhydrous K_2_CO_3_ (914.36 mg, 6.616 mmol, 4 eq.). After stirring the reaction mixture for 30 min at room temperature, ethyl chloroacetate (760.2 mg, 6.203 mmol, 3.75 eq.) was slowly added. This was followed by stirring at room temperature under a nitrogen atmosphere. The reaction was monitored by thin layer chromatography (TLC) and was halted after 7 h. The product mixture was vacuum filtered to obtain a yellow filtrate which was concentrated by a rotor vapor to obtain a yellow crude product. The crude product was purified by column chromatography using silica gel (Hexane/ethyl acetate/acetone, 1:1:1, *v/v/v*). The final product was a yellow powder, 911.73 mg, 85.25% yield. The intermediate compounds 9 and 13 were synthesized using a similar method (Scheme 2) to afford compound 9; a yellow powder, 61.0% yield and compound **13**; a yellow powder, 73.1% yield. The structures of these three compounds were confirmed using ^1^H & ^13^C NMR characterizations.

Compound **2**: δ^1^H (500 MHz, DMSO-d_6_) δ 12.42 (s, 1H), 7.81–7.74 (m, 2H), 7.11 (d, *J* = 8.7 Hz, 1H), 6.80 (d, *J* = 2.3 Hz, 1H), 6.44 (d, *J* = 2.3 Hz, 1H), 4.96 (d, *J* = 3.3 Hz, 4H), 4.89 (s, 2H), 4.84 (s, 2H), 4.23–4.14 (m, 6H), 4.12 (t, *J* = 7.1 Hz, 2H), 1.26–1.13 (m, 12H). δ^13^C (125 MHz, DMSO-d_6_) δ 178.11, 169.05, 168.92, 168.85, 168.47, 164.00, 161.36, 156.49, 155.02, 150.32, 147.06, 137.14, 123.54, 123.29, 113.98, 105.96, 93.78, 68.52, 66.06, 65.58, 65.48, 61.37, 61.27, 61.16, 61.07, 14.51.

Compound **9**: δ^1^H (500 MHz, DMSO-d_6_) δ 12.90 (s, 1H), 8.09–8.02 (m, 2H), 7.16–7.09 (m, 2H), 6.96 (s, 1H), 6.82 (d, *J* = 2.3 Hz, 1H), 6.41 (d, *J* = 2.3 Hz, 1H), 4.94 (d, *J* = 7.5 Hz, 4H), 4.19 (qd, *J* = 7.1, 4.1 Hz, 4H), 1.23 (td, *J* = 7.1, 3.7 Hz, 6H). δ^13^C (125 MHz, DMSO-d_6_) δ 182.35, 168.68, 168.40, 163.87, 163.79, 161.55, 161.08, 157.45, 128.73, 123.70, 115.50, 105.47, 104.33, 98.79, 98.73, 93.86, 65.30, 65.08, 61.24, 61.16, 14.39.

Compound **13**: δ^1^H (500 MHz, DMSO-d_6_)δ 12.89 (s, 1H), 7.70 (dd, *J* = 8.6, 2.2 Hz, 1H), 7.63 (d, *J* = 2.2 Hz, 1H), 7.13–7.04 (m, 2H), 6.79 (d, *J* = 2.3 Hz, 1H), 6.41 (d, *J* = 2.3 Hz, 1H), 4.99–4.93 (m, 6H), 4.19 (qd, *J* = 7.1, 5.5 Hz, 6H), 1.23 (td, *J* = 7.1, 4.3 Hz, 9H). δ^13^C (125 MHz, DMSO-d_6_) δ 182.61, 169.09, 168.88, 168.59, 163.99, 163.83, 161.75, 157.63, 151.16, 147.87, 124.11, 121.29, 114.41, 112.64, 105.67, 104.97, 98.95, 98.91, 94.10, 66.03, 65.73, 65.52, 61.46, 61.36, 61.23, 14.62, 14.58.

Synthesis of 2,2′-((4-(3,7-bis(carboxymethoxy)-5-hydroxy-4-oxo-4H-chromen-2-yl)-1,2-phenylene)bis(oxy))diacetic acid (**3**); 2-(4-(7-(carboxymethoxy)-5-hydroxy-4-oxo-4H-chromen-2-yl)phenoxy)acetic acid (**10**) and 2,2′-((4-(7-(carboxymethoxy)-5-hydroxy-4-oxo-4H-chromen-2-yl)-1,2-phenylene)bis(oxy))diacetic acid (**14**).

Compound **2** (300 mg, 0.464 mmol, 1 eq.) was dissolved in a solvent mixture of tetrahydrofuran (THF): water (1:3, *v/v*). Into this solution was added lithium hydroxide monohydrate (77.95 mg, 1.856 mmol, 4 eq.), followed by stirring at room temperature. The progress of the reaction was monitored by thin layer chromatography and the reaction was completed after 10 h. The unreacted lithium hydroxide monohydrate was neutralized using dilute hydrochloric acid, affording a yellow precipitate. The precipitate was filtered under vacuum to obtain a yellow crude product which was purified by washing in repeated cycles using acetone and hexane. The final product was a light-yellow powder, compound **3**, 203.3 mg, 81.99% yield. The corresponding intermediate compounds 10 and 14 were obtained using a similar method (Scheme 2) to achieve compound 10; a light-yellow solid, 96.0% yield and compound 14, a light-yellow solid, 87.8% yield. The structures of these three compounds were confirmed using ^1^H & ^13^C NMR characterizations.

Compound **3**: δ^1^H (500 MHz, DMSO-d_6_) δ 12.45 (s, 1H), 7.82 (s, 1H), 7.79–7.73 (m, 1H), 7.04 (s, 1H), 6.77–6.73 (m, 1H), 6.38 (s, 1H), 4.79 (d, *J* = 15.7 Hz, 6H), 4.73 (s, 2H). δ^13^C (125 MHz, DMSO-d_6_) δ 178.21, 170.41, 164.25, 161.26, 156.48, 154.90, 150.38, 147.16, 137.16, 122.96, 114.52, 113.54, 105.75, 98.72, 93.61, 68.59, 65.60.

Compound **10**: δ^1^H (500 MHz, DMSO-d_6_) δ 12.90 (s, 1H), 8.10–8.03 (m, 2H), 7.14–7.07 (m, 2H), 6.95 (s, 1H), 6.80 (d, *J* = 2.3 Hz, 1H), 6.39 (d, *J* = 2.3 Hz, 1H), 4.83 (d, *J* = 8.6 Hz, 4H). δ^13^C (125 MHz, DMSO-d_6_) δ 182.03, 169.82, 169.54, 163.75, 163.66, 161.18, 161.01, 157.17, 128.43, 123.15, 115.16, 105.05, 103.90, 98.55, 98.48, 93.43, 65.05, 64.76.

Compound **14**: δ^1^H (500 MHz, DMSO-d_6_) δ 12.87 (s, 1H), 7.64 (dd, *J* = 8.6, 2.1 Hz, 1H), 7.56 (d, *J* = 2.1 Hz, 1H), 7.05–6.97 (m, 2H), 6.74 (d, *J* = 2.3 Hz, 1H), 6.35 (d, *J* = 2.2 Hz, 1H), 4.83 (s, 2H), 4.79 (d, *J* = 3.1 Hz, 4H). δ^13^C (125 MHz, DMSO-d_6_) δ 182.45, 170.59, 170.32, 170.00, 164.21, 163.88, 161.56, 157.56, 151.28, 147.93, 123.60, 120.88, 113.89, 111.97, 105.41, 104.64, 98.91, 93.84, 65.83, 65.66, 65.59.

Synthesis of 2,2′-((4-(3,7-bis(2-amino-2-oxoethoxy)-5-hydroxy-4-oxo-4H-chromen-2-yl)-1,2-phenylene)bis(oxy))diacetamide (**4**); 2-(4-(7-(2-amino-2-oxoethoxy)-5-hydroxy-4-oxo-4H-chromen-2-yl)phenoxy)acetamide (**11**) and 2,2′-((4-(7-(2-amino-2-oxoethoxy)-5-hydroxy-4-oxo-4H-chromen-2-yl)-1,2-phenylene)bis(oxy))diacetamide (**15**).

Compound **3** (185 mg, 0.346 mmol, 1 eq.) was suspended in thionyl chloride, and the reaction mixture was refluxed for 5 h. The reaction mixture was filtered off to obtain quercetin acetyl chloride intermediate. The quercetin acetyl chloride intermediate was transferred into an ice-cold ammonium hydroxide solution, followed by stirring at room temperature, for 2 h. The reaction mixture was concentrated in a rotavapor to obtain the crude product, which was further purified by column chromatography using silica gel (methanol/water, 9:1, *v/v*). The product, compound **4**, was a yellow powder, 150.26 mg, 81.87% yield. Compounds **11** and **15** were synthesized following a similar method (Scheme 2) to obtain compound **11**, a brown solid, 87.23% yield and compound **15**, a brown solid, 84.32% yield. The structures of compounds **4**, **11** and **15** were confirmed using ^1^H & ^13^C NMR characterizations. The purity of each of these compounds was determined using HPLC analysis, while the mass of the compounds was determined using mass spectrometry.

Compound **4**: δ^1^H (500 MHz, DMSO-d_6_) δ 12.43 (s, 1H), 7.82–7.76 (m, 2H), 7.64–7.59 (m, 2H), 7.54–7.50 (m, 2H), 7.46 (d, *J* = 7.0 Hz, 3H), 7.41 (s, 1H), 7.12 (d, *J* = 8.5 Hz, 1H), 6.76 (d, *J* = 2.3 Hz, 1H), 6.44 (d, *J* = 2.2 Hz, 1H), 4.65–4.55 (m, 6H), 4.41 (s, 2H). δ^13^C (125 MHz, DMSO-d_6_) δ 177.97, 170.10, 170.04, 169.72, 169.15, 163.85, 160.95, 156.30, 155.33, 150.46, 147.38, 137.32, 123.30, 122.90, 114.87, 113.84, 105.62, 98.76, 93.53, 70.58, 68.27, 67.59, 67.14. Calculated molecular weight, C_23_H_22_N_4_O_11_: 530.45; found: ESI-MS: 531.29 ([M + H]^+^).

Compound **11**: δ^1^H (500 MHz, DMSO-d_6_) δ 12.90 (s, 1H), 8.07 (s, 1H), 8.02 (s, 1H), 7.63 (s, 2H), 7.47 (s, 2H), 7.13 (s, 2H), 6.96 (s, 1H), 6.78 (d, *J* = 16.6 Hz, 1H), 6.42 (s, 1H), 4.60–4.55 (m, 4H). δ^13^C (125 MHz, DMSO-d_6_) δ 182.19, 169.55, 169.22, 164.33, 163.79, 163.67, 161.52, 161.25, 157.28, 157.22, 128.77, 128.53, 123.36, 121.18, 116.18, 115.51, 105.23, 105.14, 104.11, 103.88, 103.25, 98.92, 98.84, 93.66, 93.57, 67.08, 66.88. Calculated molecular weight, C_19_H_16_N_2_O_7_: 384.34; found: ESI-MS: 385.48 ([M + H]^+^).

Compound **15**: δ^1^H (500 MHz, DMSO-d_6_) δ 12.87 (s, 1H), 7.74 (dd, *J* = 8.6, 2.2 Hz, 1H), 7.68 (d, *J* = 2.2 Hz, 1H), 7.61 (s, 1H), 7.51 (s, 1H), 7.50–7.43 (m, 4H), 7.12 (d, *J* = 8.7 Hz, 1H), 7.02 (s, 1H), 6.79 (d, *J* = 2.2 Hz, 1H), 6.43 (d, *J* = 2.2 Hz, 1H), 4.64 (s, 2H), 4.60 (d, *J* = 13.3 Hz, 4H). δ^13^C (125 MHz, DMSO-d_6_) δ 177.80, 169.89, 169.84, 169.51, 168.94, 163.68, 160.77, 156.13, 155.16, 150.28, 147.21, 137.14, 123.16, 123.08, 122.71, 114.73, 114.65, 113.69, 113.63, 105.44, 98.64, 98.53, 93.37, 93.34, 70.40, 68.09, 67.41, 66.96. Calculated molecular weight, C_21_H_19_N_3_O_9_: 457.39; found: ESI-MS: 458.41 ([M + H]^+^).

Synthesis of ethyl 2-((3,7-bis(2-amino-2-oxoethoxy)-2-(3,4-bis(2-amino-2-oxoethoxy) phenyl)-4-oxo-4H-chromen-5-yl) oxy) acetate (**5**)

In an oven dried round bottomed flask, compound **4** (85 mg, 0.160 mmol, 1 eq.) was dissolved in dry dimethylformamide, followed by the addition of anhydrous potassium carbonate (33.17 mg, 0.240 mmol, 1.5 eq.). The reaction mixture was stirred at room temperature for 15 min prior to the addition of ethyl chloroacetate (29.41 mg, 0.240 mmol, 1.5 eq.). The reaction mixture was stirred at room temperature; the progress of the reaction was monitored by thin layer chromatography and the reaction was completed after 3 h. The reaction mixture was vacuum filtered to obtain a light-yellow crude product which was purified by column chromatography, using silica gel to obtain the final product, pale- yellow solid, 87.0% yield. The structure of the product was confirmed using ^1^H & ^13^C NMR characterizations. The purity of compound **5** was determined using HPLC characterization, while the mass was determined using mass spectrometry.

Compound **5**: δ^1^H (500 MHz, DMSO-d_6_) δ 7.84 (s, 1H), 7.81–7.68 (m, 2H), 7.64 (s, 1H), 7.54 (s, 2H), 7.48 (d, *J* = 6.2 Hz, 2H), 7.42 (s, 1H), 7.12 (dd, *J* = 8.7, 4.4 Hz, 1H), 6.89–6.80 (m, 1H), 6.52 (d, *J* = 2.3 Hz, 1H), 4.92 (s, 2H), 4.63–4.53 (m, 5H), 4.27 (d, *J* = 9.9 Hz, 2H), 4.19 (p, *J* = 6.8 Hz, 3H), 1.23 (t, *J* = 7.1 Hz, 3H). δ^13^C (125 MHz, DMSO-d_6_) δ 172.69, 170.89, 170.52, 170.19, 169.53, 168.68, 162.47, 159.07, 158.44, 152.45, 150.30, 147.75, 139.97, 123.60, 123.56, 123.15, 123.09, 114.95, 114.89, 114.23, 109.35, 98.73, 98.66, 95.41, 71.14, 68.59, 67.97, 67.48, 66.14, 61.40, 14.62, 14.59. Calculated molecular weight, C_27_H_28_N_4_O_13_: 616.54; found: ESI-MS: 617.41 ([M + H]^+^).

Synthesis of 2-(2,3-dihydrobenzo[b][1,4] dioxin-6-yl)-3,5,7-trihydroxy-4H-chromen-4-one (**6**)

The synthesis of compound **6** was achieved using a modified procedure adopted from literature [41]. Quercetin, compound **1**, (400 mg, 1.323 mmol, 1 eq.) was dissolved in dry acetonitrile in an oven dried round bottomed flask. Anhydrous K_2_CO_3_ (105.18 mg, 0.761 mmol, 0.575 eq.) was added into the flask, followed by stirring at room temperature for 15 min, under a nitrogen atmosphere. Into this reaction mixture was added 1,2-dibromoethane (534.27 mg, 2.844 mmol, 2.15 eq.), dropwise. The reaction was stirred at room temperature for 45 min and the temperature of the reaction was gradually raised to 100 °C. Thin layer chromatography was used to monitor the progress of the reaction which was stopped after 4 h. The reaction mixture was vacuum filtered, and the resultant filtrate was precipitated in ice-cold water. The precipitate was filtered under vacuum and washed in repeated cycles using ice-cold water. The resultant yellow-brown powder was purified by column chromatography using silica gel (acetone/dichloromethane, 1:5, *v/v*) to afford a brown powder; compound **6**, 45.11% yield. The structure of the product was confirmed using ^1^H & ^13^C NMR characterization. The mass of this compound was determined using mass spectrometry.

Compound **6**: δ^1^H (500 MHz, DMSO-d_6_) δ 12.40 (s, 1H), 10.83 (s, 1H), 9.58 (s, 1H), 7.74–7.64 (m, 2H), 7.02 (d, *J* = 8.6 Hz, 1H), 6.46 (d, *J* = 2.0 Hz, 1H), 6.19 (d, *J* = 2.1 Hz, 1H), 4.36–4.29 (m, 4H). δ^13^C (125 MHz, DMSO-d_6_) δ 175.53, 163.57, 160.22, 155.72, 145.16, 144.57, 142.65, 135.85, 123.45, 120.66, 120.59, 116.73, 116.06, 115.99, 102.60, 97.83, 97.73, 93.13, 93.05, 63.99, 63.56. Calculated molecular weight, C_17_H_12_O_7_: 328.28; found: ESI-MS: 329.28 ([M + H]^+^).

Synthesis of 2,2′,2″-((2-(2,3-dihydrobenzo[b][1,4]dioxin-6-yl)-4-oxo-4H-chromene-3,5,7-triyl)tris(oxy))triacetamide (**7**)

Compound **6** (100 mg, 0.305 mmol, 1 eq.) was dissolved in dry dimethylformamide in an oven dried round bottomed flask. Anhydrous K_2_CO_3_ (126.45 mg, 0.915 mmol, 3 eq.) was added, followed by stirring at room temperature for 5 h. 2-chloroacetamide (99.77 mg, 1.067 mmol, 3.5 eq.) was slowly added into the reaction mixture, followed by the addition of a catalytic amount of potassium iodide. The reaction mixture was stirred at room temperature, under a nitrogen atmosphere and the progress of the reaction was monitored by thin layer chromatography for 72 h. The crude product was vacuum filtered prior to purification by column chromatography using silica gel. The final product was a yellow powder, 76.67% yield. The structure of the product was confirmed using ^1^H & ^13^C NMR characterizations. The purity of this compound was determined using HPLC analysis, while the mass of the compound was determined using mass spectrometry.

Compound **7**: δ^1^H (500 MHz, DMSO-d_6_) δ 8.08 (s, 1H), 7.74 (s, 1H), 7.64 (d, *J* = 22.9 Hz, 4H), 7.46 (d, *J* = 29.6 Hz, 2H), 7.03 (d, *J* = 8.5 Hz, 1H), 6.90 (s, 1H), 6.65 (s, 1H), 4.58 (d, *J* = 34.4 Hz, 4H), 4.39–4.23 (m, 6H). δ^13^C (125 MHz, DMSO-d_6_) δ 172.93, 170.07, 169.74, 169.02, 162.49, 157.71, 157.65, 152.67, 145.88, 143.37, 139.01, 122.76, 121.84, 117.37, 117.20, 108.42, 98.42, 94.87, 70.05, 67.77, 67.04, 64.52, 64.05. Calculated molecular weight, C_23_H_21_N_3_O_10_: 499.43; found: ESI-MS: 500.40 ([M + H]^+^).

### 2.2. High Performance Liquid Chromatography (HPLC) Characterization

The purity of the flavonoid acetamide derivatives was determined by High-performance liquid chromatography (HPLC) analysis. An Advion HPLC instrument equipped with an autosampler (Avant A-2052), dual line gradient pump (Avant A-2011), a Diode-Array Detection (DAD) UV-Vis detector (Avant A-2045) and a column compartment was used. The chromatographic separation was done on a Kromasil^®^ reverse phase C-18 HPLC column (5 µm particle size, pore size 100 Å, L × I.D. 150 mm × 4.6 mm) as the stationary phase. The mobile phase used for the analysis was water with 0.075% acetic acid (A) and acetonitrile with 0.075% acetic acid (B).

The study samples were freshly prepared in an HPLC grade water-acetonitrile solvent system. The freshly prepared samples were diluted in the HPLC grade mobile phase prior to filtration with 0.20-micron HPLC filters, Millipore Sigma (Burlington, MA, USA). The sample was injected through the autosampler. The column was equilibrated with 35% B prior to the analysis. The flow rate of the mobile phase was 1.00 × 10^3^ µL/minute. The gradient of the mobile phase was programmed separately for each sample. The gradient was set as follows for compounds **5**, **11** & **15**: 0–15 min, 35–55% B; 15–15.5 min, 55–35% B and 15.5–16 min, 35% B. In Compound **4**, the gradient was set as: 0–30 min, 35–55% B; 30–31 min, 55–35% B and 31–32 min, 35% B while in compound **7**, the gradient was programmed as: 0–5 min, 35–40% B; 5–10 min, 40–45% B, 10–30 min, 45–55%, 30–30.5 min, 55–35% B and 30.5.5–31 min, 35% B. The column was washed thrice with 35–60% B and equilibrated with 35% B between the runs. Both the chromatogram and the UV–Vis spectra were recorded during the analysis. Each sample was identified by both the HPLC chromatogram and HPLC-UV spectrum data.

### 2.3. Mass Spectrometry (MS) Characterization

The mass-to-charge ratio (*m/z*) of the samples was determined using mass spectrometry. The Finnigan LTQ^®^ spectrometer equipped with a hyperbolic segmented linear ion trap, radial ion ejection, dual conversion dynode detector, and an integrated syringe pump was used. A heated electrospray ionization (ESI) interface was used. Liquified nitrogen was used as the sheath gas at a flow rate of 9.42 and auxiliary gas at a flow rate of 24.95. The sweep gas flow rate was set at 9.92. The capillary temperature was set at 274.63 °C. The tube lens voltage was set at 114.87 V, while the capillary voltage was set at 45.85 V. The flavonoid acetamide derivatives were dissolved in LC-MS grade water- acetonitrile solvent system modified with 0.1% formic acid. The samples were introduced into the spectrometer using a 500 µL syringe by Hamilton^®^ through the integrated syringe pump, with a 10 µL/minute flow rate. The mass-to-charge (*m/z*) ratio of the samples were obtained in the positive ion mode.

### 2.4. In Vitro Bioavailability Studies

The in vitro bioavailability studies of the flavonoid acetamide derivatives were done in simulated gastric and intestinal fluids using the dialysis tubing procedure, as previously reported [40]. The procedure involves two consecutive digestion stages: phase 1, which is a 2-h pepsin-based digestion followed by phase 2, which is a 4-h pancreatin based digestion. This procedure was adopted from Chimento, Adele, et al. (2013) [42] and Grande, Fedora, et al. (2016) [43].

*Phase 1: Pepsin digestion, 2 h*

The flavonoid acetamide derivatives were dissolved in dimethyl sulfoxide (DMSO) to achieve a concentration of 1.00 × 10^−3^ M. The porcine pepsin was prepared to achieve 2.40 × 10^4^ U porcine pepsin per 1.00 × 10^−3^ L of solution. The sodium cholate solution was prepared to attain a final concentration of 2.00 *w/v*% while hydrochloric acid was prepared at a concentration of 8.50 × 10^−1^ N, for immediate use.

The pepsin digestion phase was completed in 120 min. Into an oven dried test tube, a 1.00 mL of the newly prepared porcine pepsin solution was added, followed by 300 µL of the flavonoid acetamide solution. These two solutions were mixed prior to the addition of 3.00 mL of a fresh batch of the sodium cholate solution. The resultant solution was appropriately mixed and transferred into a dialysis tubing, which was subsequently sealed on both ends. The dialysis tubing was immersed into a freshly prepared 0.85 N hydrochloric acid solution (pH 1.0). The flask and its contents were incubated in a shaking water bath at a regulated temperature of 37 ± 0.5 °C. After 120 min, 3.00 mL of the liquid in the flask was obtained for UV-Vis analysis. The dialysis tubing was carefully recovered and readied for the second stage of the experiment, phase 2. Each sample experiment was done in triplicates.

*Phase 2: Pancreatin digestion, 4 h*

The following solutions were prepared for immediate use: a solution containing 8.00 × 10^−1^ N sodium bicarbonate with 2.26 × 10^1^ g of porcine pancreatin per 1.00 mL of solution. The dialysis tubing recovered following the pepsin digestion was carefully opened on one end and for the addition of 1.10 × 10^1^ g of amylase, 1.10 × 10^1^ g of esterase and 1.30 mL of the fresh batch of the NaHCO_3_ solution. These contents were appropriately mixed, and the dialysis tubing was suitably sealed and immersed in a flask with PBS buffer (at pH 7.0). The flask was incubated for 4 h, in a shaking water bath at a regulated temperature of 37 ± 0.5 °C. At the end of the experiment, a 3.00 mL sample of the liquid in the flask was obtained for UV-Vis analysis. All sample experiments in this phase were done in triplicates.

The calibration curve for each flavonoid acetamide derivative was prepared at both pH 1.0 and pH 7.0. The triplicate samples obtained from both phase 1 and phase 2 of the digestions were characterized using UV-Vis; the data obtained was applied to the equations obtained from the calibration curves to calculate the amount of the flavonoid acetamide derivatives in the recovered samples. The percentage (%) of the recovered flavonoid acetamide derivative for each sample after both pepsin digestion (phase 1) and pancreatin digestion (phase 2) was calculated relative to the total flavonoid content used. Thus, the (%) bioavailability was determined from Equation (1) below.

(1) $$\%\;\mathrm{Bioavailability}=\frac{\mathrm{Recovered}\;\mathrm{Content}}{\mathrm{Total}\;\mathrm{Content}}\times 100$$

The total in vitro bioavailability was defined as the sum of the % bioavailability after phase 1 and % bioavailability after phase 2 of the digestion, as shown in Equation (2). The final reported in vitro bioavailability value was the average of the three trials ± the standard deviation.

(2) Total Bioavailability=phase 1 bioavailability+phase 2 bioavailability

### 2.5. Antioxidant Activity by 2,2-Diphenyl-1-Picrylhydrazyl (DPPH) Assay

The stock solutions of the flavonoid acetamide derivatives were prepared in dimethyl sulfoxide. A fresh batch of 50 µM DPPH radical solution was prepared in methanol. The DPPH free radical scavenging activities of the flavonoid acetamide derivatives were determined using our previously reported literature procedure [40,44,45].

Into an amber vial, 1.00 × 10^2^ µL solution of the flavonoid acetamide derivative in dimethyl sulfoxide was added into a 3.00 × 10^3^ µL of a fresh batch of methanolic DPPH solution. This reaction mixture was incubated, away from light, for half an hour. The absorbance of the resultant solution was read on a UV-Vis spectrophotometer, λ_max_ = 517 nm. This experiment was done across several concentrations to obtain the concentration-based antioxidant activity data which was subsequently used to calculate the relative half-maximal inhibitory concentration (IC_50_) values.

Given the observed variation of the stability of the DPPH free radical across different solvent vehicles, the stability of the DPPH free radicals was established in both methanol and dimethyl sulfoxide solvents. The percentage DPPH free radical scavenging activity of the flavonoid acetamide derivatives was computed against dimethyl sulfoxide, as per Equation (3).

(3) $$\%\;\mathrm{DPPH}\;\mathrm{RSA}=\left[\frac{\left(\mathrm{Absorbance}\;\mathrm{of}\;\mathrm{control}-\mathrm{Absorbance}\;\mathrm{of}\;\mathrm{sample}\;\right)}{\mathrm{Absorbance}\;\mathrm{of}\;\mathrm{control}}\right]\times 100$$

Absorbance control is the absorbance of the blank methanolic DPPH sample with DMSO. The absorbance values were obtained at λ_max_ = 517 nm.

The % RSA obtained was plotted and used to obtain the relative half-maximal inhibitory concentration (IC_50_) values, which were double confirmed using verified online computational tools [40,46].

### 2.6. Drug Likeness, ADME and Pre-ADMET Properties Prediction

The drug development process is marked by a myriad of time and resource consuming steps. The sequence of steps includes the determination of the physicochemical properties, pharmacokinetics, drug likeness, biological activity, and the absorption, distribution, metabolism, excretion, and toxicity (ADMET) properties. The determination of these properties plays a key role in ensuring drug conformity of the drug molecules. Owing to the time and resource cost in the determination of the drug properties, the process of drug development employs several computational tools. These tools aim at supplying precise information suitable in the preliminary evaluation of molecules as drug candidates. In this study, we used a series of computational tools to determine the drug likeness of the flavonoid acetamide derivatives as possible drug candidates. Three main computational webtools were employed in this study: SwissADME [47], the Osiris property explorer [48], and the Ames toxicity test [40].

SwissADME is a free webtool that enables one to evaluate the drug likeness, the pharmacokinetics and the medicinal affability of small molecules as drug candidates. This tool can be accessed through http://www.swissadme.ch/ (accessed on 15 August 2022). The small molecules, in this case the flavonoid acetamide derivatives, were sketched on using Chemdraw software (v21.0.0) and the sketch was transferred into the simplified molecular-input line-entry system (SMILES). The SMILES list of each compound was introduced into the Swiss drug design tool bar. The individual calculations were run, and the data required was obtained from the output computed parameter values [47,49,50].

Ames toxicity test is an online computational tool available for the determination of the toxicity of small molecules. This tool is freely available at https://preadmet.qsarhub.com/toxicity/ (accessed on 15 August 2022). The toxicity of the flavonoid acetamide derivatives was determined through six main tests. These were the Ames TA100 (+S9), Ames TA100 (−S9), Ames TA1535 (+S9), Ames TA1535 (−S9), carcinogenicity (rat) and carcinogenicity (mouse). The flavonoid acetamide derivative molecules were first sketched on the program and the toxicity of the molecules was obtained from an output file. The results were reported as either positive (implying the presence of toxic molecules or fragments) or negative (implying the absence of toxic molecules or fragments).

Osiris property explorer is an online cheminformatics prediction tool applicable in the prediction of the physicochemical and toxicological properties of small molecules. The Osiris property explorer is based on a Java applet which provides real time cheminformatics application. This application is available online at the organic chemistry. org (https://www.organic-chemistry.org/prog/peo/) (accessed on 21 August 2022) website. The molecules were fed into the program in the form of SMILES and the select computed parameters were obtained from the output file. The data obtained from this study was categorized into toxicity and drug related properties. Four toxicological measurements were done, namely tumorigenic effect, reproductive effect, irritability effect and the mutagenic effect. The toxicity properties that conform to drugs were reported as green, while those leading to undesirable properties were reported as red. Four drug related properties were also estimated in this study: the solubility, hydrophilicity, the drug likeness and the overall drug score.

## 3. Results and Discussion

### 3.1. Selective Synthesis of Flavonoid Acetamide Derivatives

We previously reported the synthesis of global flavonoid acetamide derivatives in which all the hydroxyl groups in quercetin, apigenin, fisetin, kaempferol and luteolin were converted into acetamide groups [39,40]. In this study, we report the selective structural derivatization of quercetin, apigenin and luteolin flavonoids into partial flavonoid acetamide derivatives. In the synthesis of quercetin tetra acetamide (4), apigenin di acetamide (11), and luteolin tri acetamide (15), all the hydroxyl groups were converted into acetamide groups except the one attached at C-5 on the 2-phenylchromen-4-one structure of the flavonoid structure. The structures of these flavonoid acetamide derivatives, as well as the intermediates are shown in Scheme 1 and Scheme 2.

The synthesis of these derivatives followed our previously optimized procedure [39,40]. Briefly, the synthesis was completed in a three-step procedure in which the flavonoids were first converted into esters via polyphenol esterification process to achieve the flavonoid ester intermediate. Using the conventional ester saponification process, the flavonoid ester intermediates were hydrolyzed into flavonoid carboxylic acid intermediates. The conventional amidation process was finally applied to afford the flavonoid amide derivatives via a flavonoid carboxylic acid chloride intermediate. The flavonoid ester, carboxylic acid and the final acetamide derivatives were confirmed through ^1^H & ^13^C NMR characterization.

The structures of the flavonoid ester intermediates were confirmed using both ^1^H & ^13^C NMR characterizations. Each of these derivatives had unique hydroxyl, aromatic, methylene (ether), methylene (ester), and methyl (ester) hydrogens. All the protons were identified and quantified, through ^1^H NMR integration. In compound **2**, 12 methyl (ester), 8 methylene (ester), 8 methylene (ether), 5 aromatic, and 1 hydroxyl protons were confirmed using the ^1^H NMR. Similarly, using the ^1^H NMR, 6 methyl (ester), 4 methylene (ester), 4 methylene (ether), 7 aromatic, and 1 hydroxyl protons were confirmed for compound **9** while 9 methyl (ester), 6 methylene (ester), 6 methylene (ether), 6 aromatic, and 1 hydroxyl protons were confirmed for compound **13**. The structures of these compounds were further confirmed using ^13^C NMR characterization. The ^1^H NMR spectrum of compound **2** is shown in Figure S1. The ^1^H NMR spectra of compounds **9** and **13** are shown in Figures S2 and S3, respectively.

The formation of the flavonoid acid intermediate was evident with the elimination of the -OCH_2_CH_3_ group of the flavonoid ester intermediate. Both ^1^H & ^13^C NMR characterizations were used to confirm the formation of these intermediates. Through the ^1^H NMR, the cleavage of both the methylene and the methyl ester protons was confirmed. In compound **3**, 8 methylene (ether), 5 aromatic and 1 hydroxyl protons were confirmed while 4 methylene (ether), 7 aromatic and 1 hydroxyl protons were confirmed for compound **10**. The 6 methylene (ether), 6 aromatic, and 1 hydroxyl protons were similarly confirmed for compound **14**. The ^1^H NMR spectra of compounds **3**, **10** and **14** are shown in Figures S4–S6.

The flavonoid carboxylic acid intermediates were converted into flavonoid acetamide derivatives through the amidation process. The formation of this product was confirmed using both ^1^H & ^13^C NMR characterizations. The purity of the flavonoid acetamide derivatives was determined using HPLC analysis while the mass of the derivatives was confirmed using mass spectrometry. Through the ^1^H NMR, the methylene (ether), aromatic, amide, and hydroxyl protons were confirmed. In compound **4**, 8 methylene (ether), 5 aromatic, 8 amide, and 1 hydroxyl protons were confirmed. Compound **11** had 4 methylene (ether), 7 aromatic, 4 amide, and 1 hydroxyl protons. The 6 methylene (ether), 6 aromatic, 6 amide, and 1 hydroxyl protons were confirmed for compound 15. The ^1^H NMR spectra of compounds **4**, **11** and **15** are shown in Figures S7–S9. The structures of these compounds were further confirmed using ^13^C NMR. The purity of these compounds was determined as discussed under the HPLC section. The mass of the derivatives was positively confirmed using mass spectrometry, as discussed under the mass spectrometry section.

To understand the effect of the C-5 hydroxyl group on both the bioavailability and antioxidant activity of the flavonoid acetamide derivative, compounds **4** and **5** were synthesized. We focused on the C-5 due to its influence on electron flow considering its proximity to the carbonyl group. The synthesis of compound **5** was achieved by modifying the hydroxyl group at position C-5 on the 2-phenylchromen-4-one of compound **4** into an ester derivative. This synthesis was achieved through a single synthetic step in which the C-5 hydroxyl group was replaced by an ester group. The additional methyl (ester), methylene (ester), and methylene (ether), protons were confirmed using the ^1^H NMR characterization. Thus, 3 methyl (ester), 2 methylene (ester), 10 methylene (ether), 5 aromatic, and 8 amide protons were confirmed for compound **5** from the ^1^H NMR spectrum. Further confirmation of this structure was done using the corresponding ^13^C NMR characterization. The ^1^H NMR spectrum of compound **5** is shown in Figure S10. The purity of this derivative was determined through HPLC, and the mass was confirmed as discussed in the HPLC and mass spectrometry sections.

The catechol hydrogens (at positions C-3′ and C-4′ on the 2-phenylchromen-4-one structure) in flavonoid affect both the bioavailability and the antioxidant activity of the flavonoid. To understand the extent to which the bioavailability and antioxidant activity of the corresponding flavonoid acetamide derivative is affected by the presence of these hydroxyl groups, we synthesized a derivative in which the free hydroxyl groups of the catechol are protected. A catechol protecting group was thus used to achieve an intermediate derivative, compound **6**. The ^1^H & ^13^C NMR characterizations were used to confirm the synthesis of this intermediate. Through the ^1^H NMR, the catechol protecting methylene, the hydroxyl and the aromatic protons were all confirmed thus 4 methylene, 3 hydroxyl and 5 aromatic protons were confirmed. The corresponding ^13^C NMR affirms this structure. The ^1^H NMR spectrum of compound **6** is shown in Figure S11. The mass of compound **6** was confirmed by mass spectrometry, as discussed under the mass spectrometry section.

Compound **7** was synthesized from compound **6**, as shown in Scheme 1. The hydroxyl groups at positions C-3, C-5, and C-7′ on the 2-phenylchromen-4-one structure of compound **6** were converted into acetamide groups. The formation of this derivative was confirmed using both ^1^H & ^13^C NMR characterizations. From the integrated ^1^H NMR spectrum, the 4-catechol protection methylene, 6 methylene (ether), 6 amide, and 5 aromatic protons were all confirmed. The ^1^H NMR spectrum of compound **7** is shown in Figure S12. The purity of compound **7** was determined from the HPLC analysis, and the mass of the compound was confirmed from the mass spectrometry analysis as discussed under the HPLC and mass spectrometry sections.

### 3.2. High Performance Liquid Chromatography (HPLC) Characterization Results

**Figure 1:**
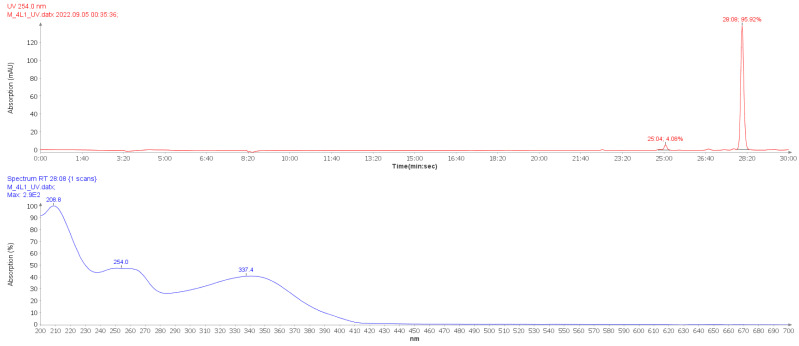
HPLC chromatogram (top) and HPLC-UV spectrum of compound **4**. The chromatogram label 28:08 indicates the sample retention time (minutes: seconds) while 95.92% indicates the percentage purity of compound **4**.

The purity of the flavonoid acetamide derivatives were analyzed using reverse phase high performance liquid chromatography, RP-HPLC. The HPLC chromatograms as well as the HPLC-UV spectra obtained were analyzed. The HPLC data confirms very high purity of the flavonoid acetamide derivatives. The percentage purity of these compounds was in the range 93.66–96.86% giving satisfactory results for biological studies. The purity of the flavonoid acetamide derivatives obtained was 95.92%, 93.66%, 96.86%, 95.11% and 95.81% for compounds **4**, **11**, **15**, **5** and **7**, respectively. The retention times of the flavonoid acetamide derivatives was obtained from the chromatogram recorded. The retention times recorded for compounds **4**, **11**, **15**, **5** and **7** were 28:08, 10:36, 12:59, 13:48 and 28:57 min: seconds, respectively. The HPLC chromatogram and HPLC- UV spectrum obtained for compound **4** is shown in Figure 1. The HPLC chromatograms of compounds **5**, **11**, **15** and **7** are shown in Figures S13–S16.

### 3.3. Mass Spectrometry (MS) Characterization Results

**Figure 2:**
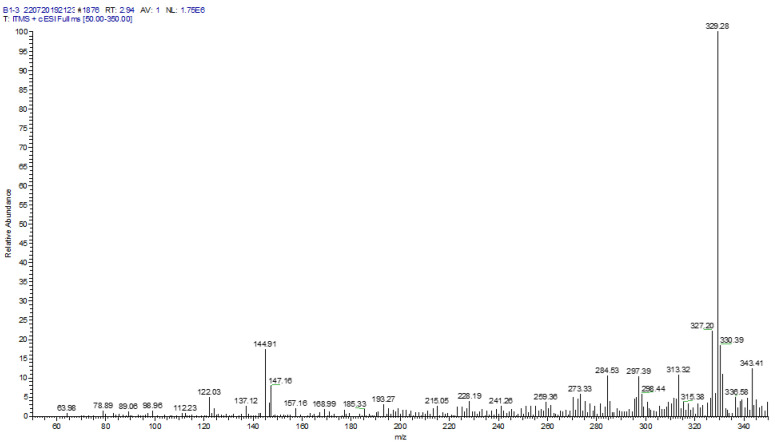
Mass spectrum of compound **6**. Calculated molecular weight, C_17_H_12_O_7_: 328.28; found: ESI-MS: 329.28 ([M + H]^+^).

The mass-to-charge ratios (*m/z*) of the flavonoid acetamide derivatives were determined using Thermo Finnigan LTQ mass spectrometer. The *m/z* values of the flavonoid acetamide derivatives were obtained using ESI-MS in the positive mode. The exact molecular weights, as determined from the *m/z* values of these compounds were reported in the form of ESI-MS ([M + H]^+^) peaks. Thus the exact molecular weights of the compounds are reported as: compound **6**: calculated molecular weight: 328.28; found: ESI-MS: 329.28 ([M + H]^+^); compound **4**: calculated molecular weight: 530.45; found: ESI-MS: 531.29 ([M + H]^+^); compound **11**: calculated molecular weight: 384.34; found: ESI-MS: 385.48 ([M + H]^+^); compound **15**: calculated molecular weight: 457.39; found: ESI-MS: 458.41 ([M + H]^+^); compound **5**: calculated molecular weight: 616.54; found: ESI-MS: 617.41 ([M + H]^+^) and compound **7**: calculated molecular weight: 499.43; found: ESI-MS: 500.40 ([M + H]^+^). A sample mass spectrum of compound **6** is shown in Figure 2. The mass spectra of compounds **4**, **11**, **15**, **5** and **7** are shown in Figures S17–S21.

### 3.4. In vitro Bioavailability Studies

Chemical modification of molecules aims at replacing select chemical moieties with other functional groups for the realization of more desired properties. In these studies, we aimed at improving the bioavailability of the flavonoids through chemical modifications of the flavonoid’s hydroxyl groups into acetamide groups. The structures of the flavonoids and the flavonoid acetamide derivatives are shown in Scheme 1 and Scheme 2. This conversion aimed at inducing tunable flexibility, pH, polarity, enzymatic stability, as well as the lipophilicity of the flavonoids, which are among the fundamental properties influencing the bioavailability of molecules [40].

The bioavailability of the flavonoid acetamides was determined using our previously reported in vitro dialysis tubing procedure [40]. The in vitro bioavailability determination was done in three parts: in vitro digestion, UV-Vis analysis and bioavailability calculation. The in vitro digestion was done using the dialysis tubing procedure, as discussed under the procedure section. The process involves two phases notably phase one and phase two during which pepsin and pancreatin digestions take place, respectively. The two phases of digestion take place consecutively under controlled pH and temperature environments. The samples obtained after each experimental phase were characterized by UV-Vis; the absorbance data and the calibration curves were used to calculate the amount of the flavonoid acetamide derivatives recovered after each phase of digestion. The bioavailability values after each stage of digestion were expressed as a percentage of the total sample and the values obtained from the three trials were expressed as an average ± standard deviation. The total percentage bioavailability of the flavonoid acetamide derivative was the sum of the percentage bioavailability after phase 1 and phase 2 of the digestion.

**Figure 3:**
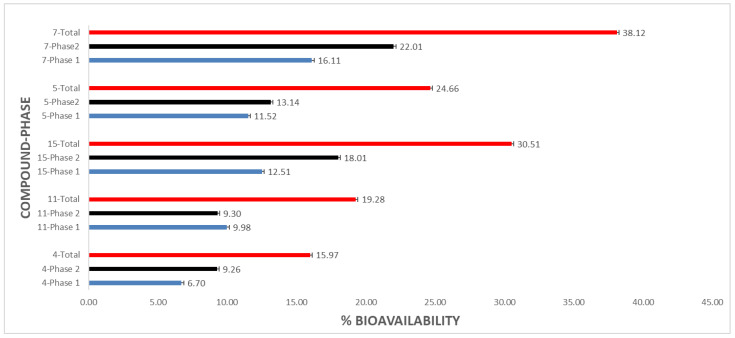
Percentage bioavailability of flavonoid acetamide derivatives after phase 1 & phase 2 digestions, and total in vitro bioavailability. Phase 1-pepsin digestion; phase 2-pancreatin digestion. The error bars show the standard deviation of the population.

We previously reported the in vitro bioavailability of five flavonoids and their corresponding flavonoid acetamide derivatives [40]. The structures of these flavonoids and flavonoid acetamide derivatives are shown in Figure S22. In this study, we report the bioavailability of the partially derived flavonoid acetamide derivatives. After the pepsin digestion, phase 1, compound **4** showed an in vitro bioavailability of 6.70 ± 0.03% and 9.26 ± 0.05% after the pancreatin digestion, phase 2. The corresponding total in vitro bioavailability was 15.97 ± 0.04%. Similarly, the in vitro bioavailability of the other flavonoid acetamide derivatives after phase 1 and phase 2 digestions were: compound **11** (9.98 ± 0.10%, 9.30 ± 0.09%); compound **15** (12.51 ± 0.02%, 18.01 ± 0.26%); compound **5** (11.52 ± 0.02%, 13.14 ± 0.01%) and compound **7** (16.11 ± 0.11%, 22.01 ± 0.21%). The corresponding total in vitro percentage bioavailability for the compounds **11**, **15**, **5** and **7** were: 19.28 ± 0.19%, 30.51 ± 0.27%, 24.66 ± 0.01% and 38.12 ± 0.16%, respectively. The percentage bioavailability values recorded for compounds **4**, **11**, **15**, **5** and **7** are graphically represented in Figure 3.

The partially modified flavonoid acetamide derivatives showed enhanced in vitro bioavailability compared to the unmodified flavonoids. The in vitro bio availabilities of the unmodified flavonoids were reported in our previous work [40]. The difference in the bioavailability values between the unmodified flavonoids and the flavonoid acetamide derivatives can be explained by the differences in the pH value, solubility, polarity, enzyme specificity and the lipophilicity between these compounds. The higher bioavailability values of the compounds **4**, **11** and **15** relatives to the unmodified flavonoids (compounds **1**, **8** and **12**, respectively) can be explained by the enhanced lipophilicity in these compounds as a result of the methylene groups of the acetamide derivatives. The structure of the flavonoid and flavonoid acetamide derivative plays a key role in determining the overall bioavailability of the molecule. The partially derivatized flavonoid acetamide derivatives showed slightly reduced bioavailability relative to the fully derivatized flavonoid acetamides. The structures of the fully derivatized flavonoids derived from compounds **1**, **8** and **12** are shown in Figure S22 as compounds 1S3, 2S3 and 5S3, respectively. The in vitro bioavailability of the globally derivatized flavonoid acetamide derivatives were reported in our previous work [40]. The slight drop in the bioavailability can be explained by the reduction in the lipophilicity of the molecule when the acetamide group at the C-5 on the 2-phenylchromen-4-one structure of the molecule is replaced by a hydroxyl group.

Compound **5** showed a higher bioavailability than compound **4**. The difference between compounds **5** and **4** is the addition of the methylene and methyl groups at the C-5 position on the 2-phenylchromen-4-one structure. These groups are highly lipophilic and could explain the enhanced bioavailability observed in compound **5**. The catechol protection of the quercetin molecule at position C-3′ and C-4′ on the 2-phenylchromen-4-one structure yields compound **6**. The conversion of the hydroxyl groups at positions C-3, C-5 and C-7 on the 2-phenylchromen-4-one structure into acetamide groups afforded compound **7**. The total in vitro bioavailability of compound **7** was 38.12%, a significantly higher value than that recorded for the unmodified flavonoid, compound **1** [40]. The additional methylene groups on compound **7** could account for the difference in bioavailability recorded.

### 3.5. Antioxidant Activity by 2,2-Diphenyl-1-Picrylhydrazyl (DPPH) Assay Results

The 2,2-diphenyl-1-picrylhydrazyl (DPPH) assay was used to evaluate the free radical scavenging capacity of the flavonoid acetamide derivatives. This assay is based on the colorimetric quantification of the DPPH free radicals. The reaction of the DPPH radicals with an antioxidant compound results in the loss of the DPPH free radical properties. The loss of the DPPH free radical properties is identifiable through a color change; a discoloration marked by the formation of a yellow color from the initial violet color. Slight antioxidant activity may be visually imperceptible; such a change may still be monitored spectrophotometrically through UV-Vis analysis at λ_max_ = 517 [40,44,45].

The ability of the flavonoid acetamide derivatives to scavenge the DPPH free radicals is influenced by the concentration of both the flavonoid acetamide derivative and the DPPH free radicals, the reaction time, and presence of light during the reaction. The reaction time was set at 30 min and the concentration of the DPPH free radicals was 55 µM. The concentration of the flavonoid acetamide derivatives was varied and the experiment was carried out in the dark. The actual concentration of the DPPH free radical before the addition of the flavonoid acetamide derivative was used as the standard for the purposes of the estimation of the DPPH free radical scavenging effect. The decrease in the DPPH free radical concentration upon the addition of the flavonoid acetamide derivative was used to calculate the antioxidant activity of the flavonoid acetamide derivatives. Thus, the greater the decrease in the DPPH free radical concentration, the higher the antioxidant activity. The antioxidant activity was expressed as the % RSA. The final antioxidant activity was expressed as the relative IC_50_ value; the 50% of the maximal inhibitory concentration of the flavonoid acetamide derivatives. Since the IC_50_ denotes the concentration of the flavonoid acetamide derivative required to achieve half the maximal DPPH free radical inhibitory activity, the IC_50_ is inversely proportional to the antioxidant activity thus the flavonoid acetamide derivative with the highest antioxidant activity will have the lowest IC_50_ value [40].

**Figure 4:**
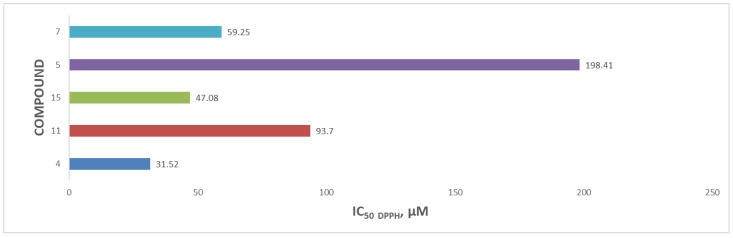
DPPH free radical scavenging activity of the flavonoid acetamide derivatives expressed as IC_50_ values.

The relative IC_50_ values for the flavonoid acetamide derivatives were in the range 31.52–198.41 µM. Compound **4** had the highest antioxidant activity, while compound **5** had the lowest antioxidant activity. The IC_50_ values recorded for compounds **4**, **11**, **15**, **5** and **7** were 31.52 µM, 93.70 µM and 47.08 µM, 198.41 µM, and 59.25 µM, respectively. The corresponding unmodified flavonoids from which compounds **4**, **11** and **15** were obtained are shown in Figure S22. Each of these three compounds had lower IC_50_ values than the partially derivatized flavonoid acetamide derivatives [40]. The improved antioxidant activities in these unmodified flavonoids can be explained by the presence of the hydroxyl groups at position C-5 on the 2-phenylchromen-4-one structure of the compounds, which take part in the scavenging of the DPPH free radicals. We previously reported the antioxidant activities of the globally modified flavonoid acetamide derivatives in which all the hydroxyl groups in the unmodified flavonoids were converted into the acetamide group. The antioxidant activities reported in the global flavonoid acetamide derivatives were lower than those of the unmodified flavonoids [40]. The antioxidant capacities of the flavonoid acetamide derivatives, expressed as the DPPH free radical inhibition IC_50_ values, are shown in Figure 4.

The antioxidant activities of the partially derivatized flavonoid acetamide derivatives, compounds **4**, **11** and **15**, were all higher than those of the corresponding globally derivatized flavonoid acetamide derivatives, compounds 1S3, 2S3 and 5S3, respectively. The partially derived flavonoid acetamide derivatives all have the same functional groups as their corresponding global flavonoid acetamide derivatives, except that in the partial flavonoid acetamide derivatives, the C-5 hydroxyl group on the 2-phenylchromen-4-one structure of the flavonoid is unmodified. The presence of the unmodified hydroxyl group at C-5 on the 2-phenylchromen-4-one structure therefore explains the difference in the antioxidant activity of these compounds.

Compound **5** is structurally similar to compound **4**, save for the additional methylene and methyl group at position C-5 on the 2-phenylchromen-4-one structure of the molecule. The methylene and methyl groups do not enhance the scavenging of free radicals and therefore can be attributed to the decrease in the antioxidant activity. The catechol hydroxyl groups attached to the positions C-3′ and C-4′ on the 2-phenylchromen-4-one structure of the 7 have been protected using catechol protecting groups, lipophilic methylene groups. The methylene group in the protection group disrupts the electron flow, thus negatively affecting the ability of this compound to scavenge the free radicals. The antioxidant of compound **7** is further limited by the presence of the methylene groups in the three acetamide groups. This accounts for the minimal antioxidant activity of this compound.

### 3.6. Structure-Activity Relationship on the Bioavailability and Antioxidant Properties

The differences in the bioavailability of the unmodified flavonoids (compounds **1**, **8** & **12**), the partial flavonoid acetamide derivatives (compounds **4**, **11** & **15**) and the global flavonoid acetamide derivatives (compounds 1S3, 2S3 & 5S3, Figure S22) can be explained by their different structures and hence physicochemical properties of these molecules. The unmodified flavonoids had the lowest bioavailability compared to both the globally and partially derivatized flavonoid acetamide derivatives. The key structural differences between the unmodified flavonoids and the flavonoid acetamide derivatives are the groups attached to the 2-phenylchromen-4-one structures. In the global flavonoid acetamide derivatives, all the hydroxyl groups of the flavonoids are replaced by the acetamide group. The acetamide group consists of lipophilic sp3 hybridized methylene groups which a play key role in the enhancement of the overall bioavailability of the flavonoid acetamide derivative. In the partially derived flavonoid acetamide derivatives, all but the hydroxyl group attached to position C-5 on the 2-phenylchromen-4-one are modified into acetamide groups. The introduction of these groups could account for the enhanced bioavailability of these flavonoid acetamide derivatives relative to the unmodified flavonoids. It was observed that the fully derivatized flavonoids had higher bioavailability than the corresponding partially derivatized flavonoids. Considering that the only difference between the partially derivatized and the global flavonoid acetamide derivatives is the group attached to the C-5 on the 2-phenylchromen-4-one structure, then such differences in the bioavailability may be attributed to the structural differences. Compound **5** showed a higher bioavailability than compound **4**, from which it was derived. The hydroxyl group in compound **4** was replaced by an ester group, which has sp3 hybridized lipophilic methylene and methyl groups, to afford compound **5**. Thus, the ester group accounts for the increase in the bioavailability between these two compounds. A similar observation was made for compound **7**. The replacement of the hydroxyl group with an acetamide group led to an increase in bioavailability.

A structure-activity review of the observed antioxidant activity of these flavonoids indicated that the presence of hydroxyl groups was associated with high antioxidant activity, while the increase in the lipophilic groups such as the sp^3^ hybridized methylene and methyl groups was associated with low antioxidant activity. This could be due to the disruption in the electron flow, an important process in the scavenging of the DPPH free radicals. Whereas the hydroxyl group at C-5 on the 2-phenylchromen-4-one structure of the flavonoid is sterically hindered by the carbonyl group, it still plays a significant role in the free radical scavenging and hence the antioxidant capacity of the flavonoids. This conclusion can be drawn from the observed differences in the DPPH free radical scavenging activity of the partially and globally derived flavonoid acetamide derivatives. The globally derived flavonoids, compounds 1S3, 2S3 and 5S3 (Figure S22), only differ with the partially derived flavonoids, compounds **4**, **11** and **15** on the groups attached to the C-5 on the 2-phenylchromen-4-one structure of the flavonoid. The globally derived flavonoids have an acetamide group while the partially derived flavonoid acetamide derivatives have a hydroxyl group at the C-5 on the 2-phenylchromen-4-one structure of the flavonoid. The acetamide group has disrupted electron flow and compared to the hydroxyl group, which supports electron delocalization along the flavonoid rings. Compounds **5** and **7** have lower antioxidant properties compared to the flavonoid from which they were derived, compound **1**. Compound **5** has an ester group at position C-5 and 4 acetamide groups at positions C-3′, C-4′, C-3 & C-7 on the 2-phenylchromen-4-one structure. Compound **7** has three acetamide groups at positions C-3, C-5 & C-7 and a catechol protecting group at positions C-3′ and C-4′ on the 2-phenylchromen-4-one structure. The presence of the methylene groups in compounds **5** and **7** disrupt the free electron delocalization and thus could explain the low antioxidant capacities observed in these compounds.

### 3.7. Drug Likeness, ADME and Pre-ADMET Properties Results

The physicochemical properties of flavonoids play a critical role in the estimation of the overall biological activity and the effectiveness of drug molecules. These properties vary depending on the structure of the molecule under study. Several structure activity relationships of different drug molecules can be estimated from the prevalent physicochemical properties. The absorption, distribution, metabolism, excretion, and toxicity (ADMET) properties of molecules rely heavily on these properties [51,52,53].

The main physical properties of molecules include the number of rotatable bonds, the number of hydrogen bond acceptors, number of hydrogen bond donors, and the topological polar surface area (TPSA) of the molecules. In addition, the lipophilicity of the molecule as determined from the calculated partition coefficient, cLogP octanol/water, values is an important property in the evaluation of the drug relevance and drug likeness of a molecule. Pharmacokinetic properties of drugs, such as the gastrointestinal absorption play a key role in the overall applications of drug molecules. The global drug likeness of candidate drug molecules can be estimated from the Lipinski’s rule of five.

The physicochemical, lipophilicity and solubility properties are very crucial properties in the determination of the oral bioavailability of drug candidates. The key physicochemical properties in the estimation of the oral bioavailability of drugs are the molecular weight, polarity, and the flexibility of the molecule. Based on Swiss ADME, molecules with good oral bioavailability have molecular sizes in the range of 150–500 amu. Compounds **11**, **15** and **7** had molecular weights within this range, which are positive indicators of good oral bioavailability. The polarity of a molecule can be measured by the TPSA, while the solubility of a molecule can be estimated by the Log S (ESOL) values. Molecules with a TPSA of 20–130 Å^2^ and Log S (ESOL) of −6.0–0.0 may have favorable oral bioavailability. All the compounds in this study had a TPSA greater than 130 Å^2^ and a Log S (ESOL) within the −6.0–0.0 range. The latter indicates positive oral bioavailability properties. The flexibility of a molecule as estimated from the number of rotatable bonds is a paramount factor in the determination of the oral bioavailability of molecules. Molecules with 0–9 rotatable bonds have favorable oral bioavailability. Compound **11** had **7** rotatable bonds while the other compounds had more than 9 rotatable bonds.

The Lipinski’s rule of five is used to largely predict the oral bioavailability of a drug molecule based on physicochemical properties. Based on this rule, molecules with good oral bioavailability have not more than 5 hydrogen bond donors, not more than 10 hydrogen bond acceptors, molecular weights smaller than 500 and a lipophilicity, measured by the cLogP, not greater than 5 [54]. Based on these properties, compound **11** has zero violations, compounds **15** and **7** each have one violation, while compounds **4** and **5** each have 2 violations of the Lipinski’s rule of 5. Whereas each of the above discussed properties are important in the determination of the oral bioavailability and hence the drug likeness of a molecule, few drug molecules satisfy these properties. The calculated physicochemical properties of the flavonoid acetamide derivatives in this study are shown in Table 1 below.

The determination of the toxicity of compounds is a long, tedious process that can be realized using computational tools. The Ames toxicity test enables the identification of compounds based on their mutagenicity. Based on this test, compounds can be classified as being either mutagenic or non-mutagenic. Mutagenic compounds will be classified as positive, while non-mutagenic compounds would be classified as being negative for the mutagenicity test. In this study, four mutagenic tests were performed. These are the Ames TA100 (+S9), Ames TA100 (−S9), Ames TA1535 (+S9), and Ames TA1535 (−S9). All the flavonoid acetamide derivatives in this study tested negative for all the four mutagenicity tests. The carcinogenic effects of the flavonoid acetamide derivatives were further tested using the Ames test. Two carcinogenicity tests of the flavonoid acetamide derivatives were done: carcinogenicity (mouse) and carcinogenicity (rat). The carcinogenic effect was reported as either positive or negative where the positive carcinogenicity result denotes the possibility of carcinogenic effect. In all the flavonoid acetamide derivatives, negative carcinogenic (rat) was recorded. All the carcinogenic (mouse) results were negative save for the compounds **11** and **15**. Using the Osiris property explorer results discussed in this study, we can relate the source of the possible carcinogenic effects of the compounds **11** and **15** to the fragments of these two compounds. The predicted pre-ADMET toxicity results of the synthesized flavonoid acetamide derivatives are shown in Table 2.

The main factors considered in the drug discovery process include the determination of the prevalent biological properties and the toxicity level of the molecules. Numerous computational tools have proven useful in the estimation of the biological activity, ADME properties, as well as the toxicity level of different molecules. The Osiris property explorer is a widely used computational tool which enables chemists to calculate the on-the-fly drug relevant properties from the valid chemical structures of the molecules. This tool is exceptionally useful in the calculation of the drug likeness, the assessment of the toxicity level and the prediction of the solubility values of different molecules in the drug discovery process. In the assessment of the toxicity level of the drug molecules, the different results are color ranked as green, for molecules that conform to good drug comportment and red, for molecules that have elevated undesired risks [40,55,56].

In this study, the Osiris property explorer was used in the assessment of the drug likeness and the estimation of the toxicity level of the flavonoid acetamide derivatives. The toxicity level was assessed based on the mutagenic effect, irritant effect, reproductive effect, and tumorigenicity properties. All the flavonoid acetamide derivatives had drug conforming properties based on the tumorigenicity and irritant effects. Every compound but **11** showed drug conforming reproductive effects. The non-conforming reproductive effects associated with compound **11** do not originate from compound **11** but originate from its fragments. All the compounds but compounds **11** and **15** were associated with an undesired mutagenic effect. The undesired mutagenic effect is associated with some fragments from compounds **4**, **5** and **7**. A similar observation was made with quercetin (compound **1**), the unmodified flavonoid from which compounds **4**, **5** and **7** were derived [40]. The toxicity prediction data of the flavonoid acetamide derivatives are shown in Table 3.

In addition to the toxicity prediction, the Osiris property explorer also facilitates the prediction of drug relevant properties. Such properties include the calculated logarithm partition coefficient (cLogP), drug likeness, solubility and the overall drug score. The cLogP is a measure of the hydrophilicity of the compound. This is an important parameter since the hydrophilicity of a molecule determines the absorption of the compound. Low hydrophilicities suggest high cLogP, which are associated with poor absorption. All the flavonoid acetamide derivatives had cLogP values smaller than 5.00, the maximum cLogP value for which the compounds would show good absorption. The drug score values recorded for the flavonoid acetamide derivatives were in the range 0.18–0.71 where compound **5** had the smallest drug score value, while compound **15** had the highest drug score value. The drug relevant properties for all the flavonoid acetamide derivatives are shown in Table 3.

## 4. Conclusions

We designed, synthesized, and characterized partial flavonoid acetamide derivatives of apigenin, quercetin and luteolin. These flavonoid acetamide derivatives are 2,2′-((4-(3,7-bis(2-amino-2-oxoethoxy)-5-hydroxy-4-oxo-4H-chromen-2-yl)-1,2-phenylene)bis(oxy))diacetamide or quercetin tetra acetamide (**4**), 2-(4-(7-(2-amino-2-oxoethoxy)-5-hydroxy-4-oxo-4H-chromen-2-yl)phenoxy)acetamide or apigenin di acetamide (**11**), 2,2′-((4-(7-(2-amino-2-oxoethoxy)-5-hydroxy-4-oxo-4H-chromen-2-yl)-1,2-phenylene)bis(oxy))diacetamide or luteolin tri acetamide (**15**), ethyl 2-((3,7-bis(2-amino-2-oxoethoxy)-2-(3,4-bis(2-amino-2-oxoethoxy)phenyl)-4-oxo-4H-chromen-5-yl)oxy)acetate (**5**), and 2,2′,2″-((2-(2,3-dihydrobenzo[b][1,4]dioxin-6-yl)-4-oxo-4H-chromene-3,5,7-triyl)tris(oxy))triacetamide (**7**). The structural confirmation of these compounds was done using ^1^H & ^13^C NMR. The purity of these compounds was determined by RP-HPLC, while mass of the compounds was determined by ESI-MS. The in vitro bioavailability of the flavonoid acetamide derivatives was determined using the dialysis tubing procedure, while the antioxidant capacity was determined using the DPPH free radical scavenging activity assay. The modification of the flavonoids into flavonoid acetamide derivatives improves bioavailability, while lessening the antioxidant capacity. The structure-activity relationship studies imply that the methylene groups in the flavonoid acetamide derivatives are essential in the enhancement of the bioavailability, while the hydroxyl group plays a key role in the enhancement of the antioxidant capacity of flavonoids. The flavonoid acetamide derivatives in this study showed excellent drug likeness and low toxicity and could be further studied to understand the predominant biological applications.

## Figures and Tables

**Table 1:** Physicochemical properties of the flavonoid acetamide derivatives, based on Swiss ADME.

Compound	MW	TPSA (Å²)	R.B	H.A	H.D	L.V	Log S (ESOL)
4	530.44	259.72	13	11	5	2	−2.11
11	384.34	155.08	7	7	3	0	−3.17
15	457.39	207.40	10	9	4	1	−2.75
5	616.53	275.02	18	13	4	2	−2.31
7	499.43	205.63	10	10	3	1	−2.51

Key: TPSA—topological polar surface area, R.B—number of rotatable bonds, L.V—number of Lipinski rule of 5 violations, H.A—number of hydrogen bond acceptors, H.D—number of hydrogen bond donors, MW—Molecular weight, Log S (ESOL)—Estimated solubility.

**Table 2:** Predicted pre-ADMET results of flavonoids acetamide derivatives from Ames test.

Compounds	Toxicity Prediction	Name of Test	Result
**4**	Ames	Ames TA100 (+S9)	Negative
		Ames TA100 (−S9)	Negative
		Ames TA1535 (+S9)	Negative
		Ames TA1535 (−S9)	Negative
	Carcinogenicity	Carcinogenicity (Mouse)	Negative
		Carcinogenicity (Rat)	Negative
**11**	Ames	Ames TA100 (+S9)	Negative
		Ames TA100 (−S9)	Negative
		Ames TA1535 (+S9)	Negative
		Ames TA1535 (−S9)	Negative
	Carcinogenicity	Carcinogenicity (Mouse)	Positive
		Carcinogenicity (Rat)	Negative
**15**	Ames	Ames TA100 (+S9)	Negative
		Ames TA100 (−S9)	Negative
		Ames TA1535 (+S9)	Negative
		Ames TA1535 (−S9)	Negative
	Carcinogenicity	Carcinogenicity (Mouse)	Positive
		Carcinogenicity (Rat)	Negative
**5**	Ames	Ames TA100 (+S9)	Negative
		Ames TA100 (−S9)	Negative
		Ames TA1535 (+S9)	Negative
		Ames TA1535 (−S9)	Negative
	Carcinogenicity	Carcinogenicity (Mouse)	Negative
		Carcinogenicity (Rat)	Negative
**7**	Ames	Ames TA100 (+S9)	Negative
		Ames TA100 (−S9)	Negative
		Ames TA1535 (+S9)	Negative
		Ames TA1535 (−S9)	Negative
	Carcinogenicity	Carcinogenicity (Mouse)	Negative
		Carcinogenicity (Rat)	Negative

**Table 3:** Drug relevant and toxicity prediction scores of flavonoid acetamide derivatives from Osiris property explorer.

Compound	Toxicity				Drug-Relevant Properties			
	Irritant Effect	Reproductive Effect	Tumorigenicity	Mutagenic Effect	cLogP	Drug-Likeness	Solubility	Drug Score
**4**	Green	Green	Green	Red	−2.33	1.57	−2.96	0.36
**11**	Green	Red	Green	Green	0.35	2.71	−3.19	0.48
**15**	Green	Green	Green	Green	−0.99	1.95	−3.06	0.71
**5**	Green	Green	Green	Red	−2.09	−2.36	−3.48	0.18
**7**	Green	Green	Green	Red	−0.66	−5.47	−3.57	0.20

Green color indicates no risk or low risk, red color indicates more toxicity risk.

## Data Availability

The data presented in this study is provided in the Supplementary Materials.

## Supplementary Materials

The following supporting information can be downloaded at: https://www.mdpi.com/article/10.3390/molecules27238133/s1, Figure S1: ^1^H NMR spectrum (500 MHz, DMSO-d_6_): compound **2**; Figure S2: ^1^H NMR spectrum (500 MHz, DMSO-d_6_): compound **9**; Figure S3: ^1^H NMR spectrum (500 MHz, DMSO-d_6_): compound **13**; Figure S4: ^1^H NMR spectrum (500 MHz, DMSO-d_6_): compound **3**; Figure S5: ^1^H NMR spectrum (500 MHz, DMSO-d_6_): compound **10**; Figure S6: ^1^H NMR spectrum (500 MHz, DMSO-d_6_): compound **14**; Figure S7: ^1^H NMR spectrum (500 MHz, DMSO-d_6_): compound **4**; Figure S8: ^1^H NMR spectrum (500 MHz, DMSO-d_6_): compound **11**; Figure S9: ^1^H NMR spectrum (500 MHz, DMSO-d_6_): compound **15**; Figure S10: ^1^H NMR spectrum (500 MHz, DMSO-d_6_): compound **5**; Figure S11: ^1^H NMR spectrum (500 MHz, DMSO-d_6_): compound **6**; Figure S12: ^1^H NMR spectrum (500 MHz, DMSO-d_6_): compound **7**; S13: HPLC chromatogram of compound **5**; S14: HPLC chromatogram of compound **11**; S15: HPLC chromatogram of compound **15**; S16: HPLC chromatogram of compound **7**; Figure S17: Mass spectrum of compound **4**; Figure S18: Mass spectrum of compound **11**; Figure S19: Mass spectrum of compound **15**; Figure S20: Mass spectrum of compound **5**; Figure S21: Mass spectrum of compound **7** and Figure S22: Structure of compounds 1S0–5S0 & 1S3–5S3.
