# Supplementary material for: Selective Structural Derivatization of Flavonoid Acetamides Significantly Impacts Their Bioavailability and Antioxidant Properties
Source: Molecules. 2022 Nov 22;27(23):8133. doi: 10.3390/molecules27238133 (PMC9741454; doi:10.3390/molecules27238133)
Supplement: Supplementary file 1 [file molecules-27-08133-s001.zip › molecules-1981007-supplementary.pdf]

molecules

Article

Selective Structural Derivatization of Flavonoid Acetamides Significantly Impacts their Bioavailability and Antioxidant Properties

Daniel Kasungi Isika [1], Omowunmi A. Sadik [1,*]

[1] Department of Chemistry and Environmental Science, BioSensor Materials for Advanced Research & Technology (BioSMART Center), New Jersey Institute of Technology, 161 Warren Street, University Heights, Newark, NJ, 07102, USA.
* Correspondence: Omowunmi A. Sadik (sadik@njit.edu)

Supporting Information- SI

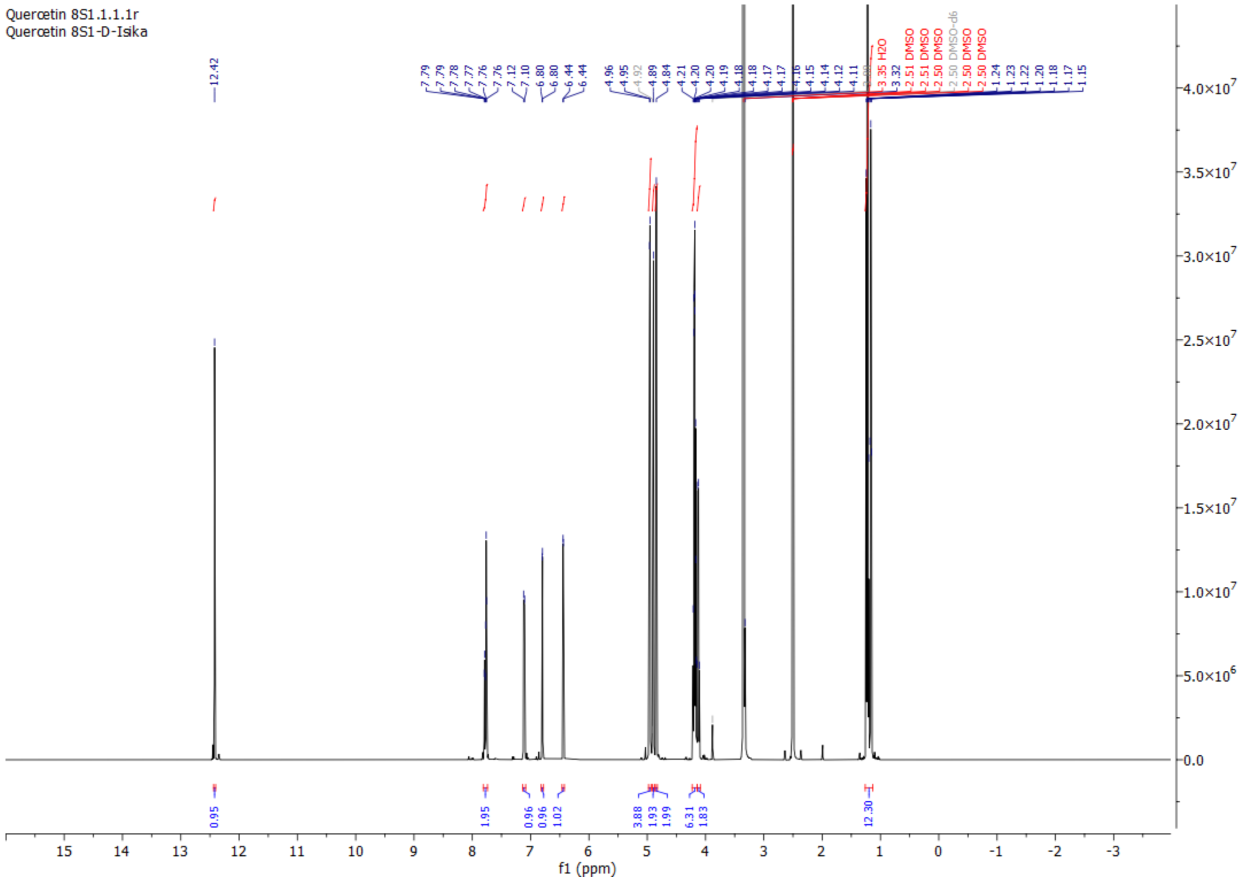

Figure S1: ^{1}H NMR spectrum (500 MHz, DMSO-d_6): compound 2

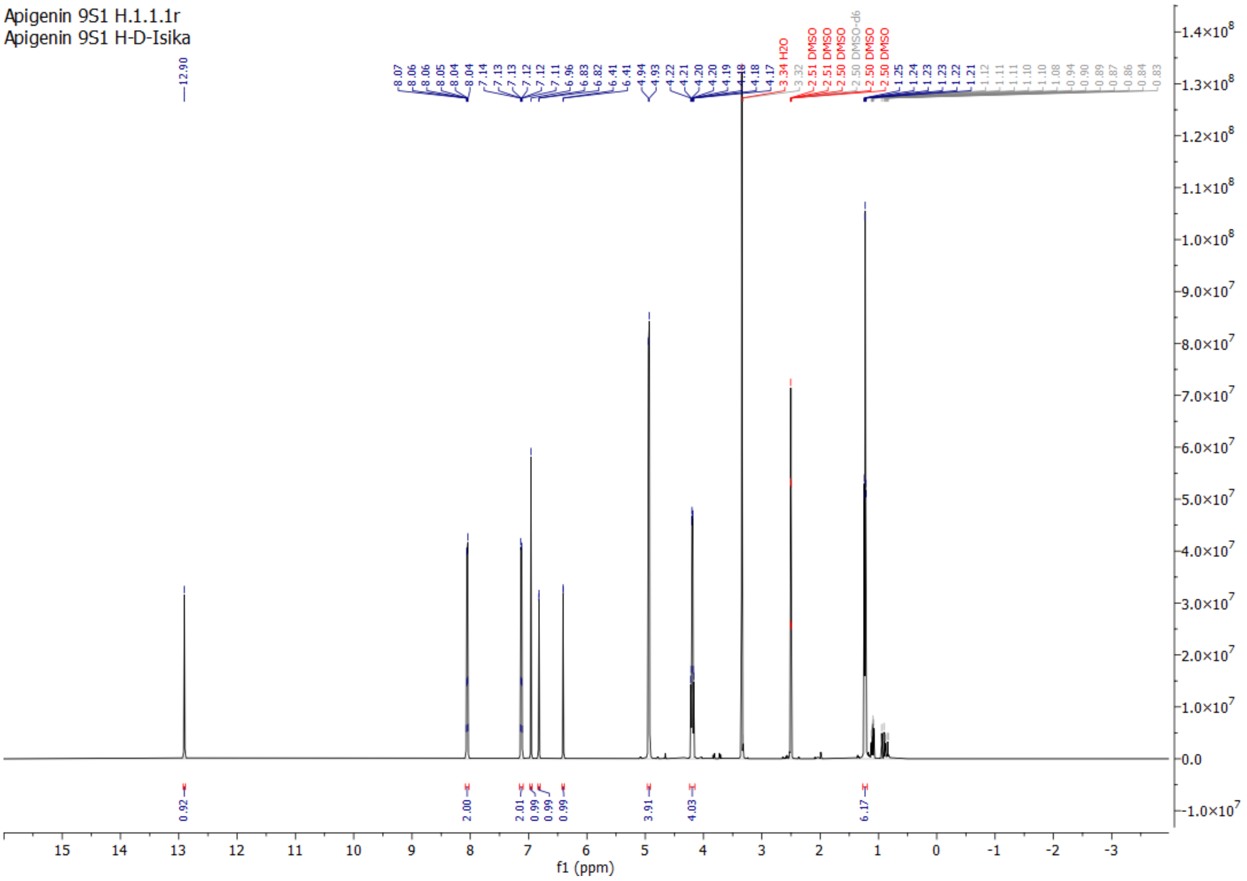

Figure S2: ^{1}H NMR spectrum (500 MHz, DMSO-d_6): compound 9

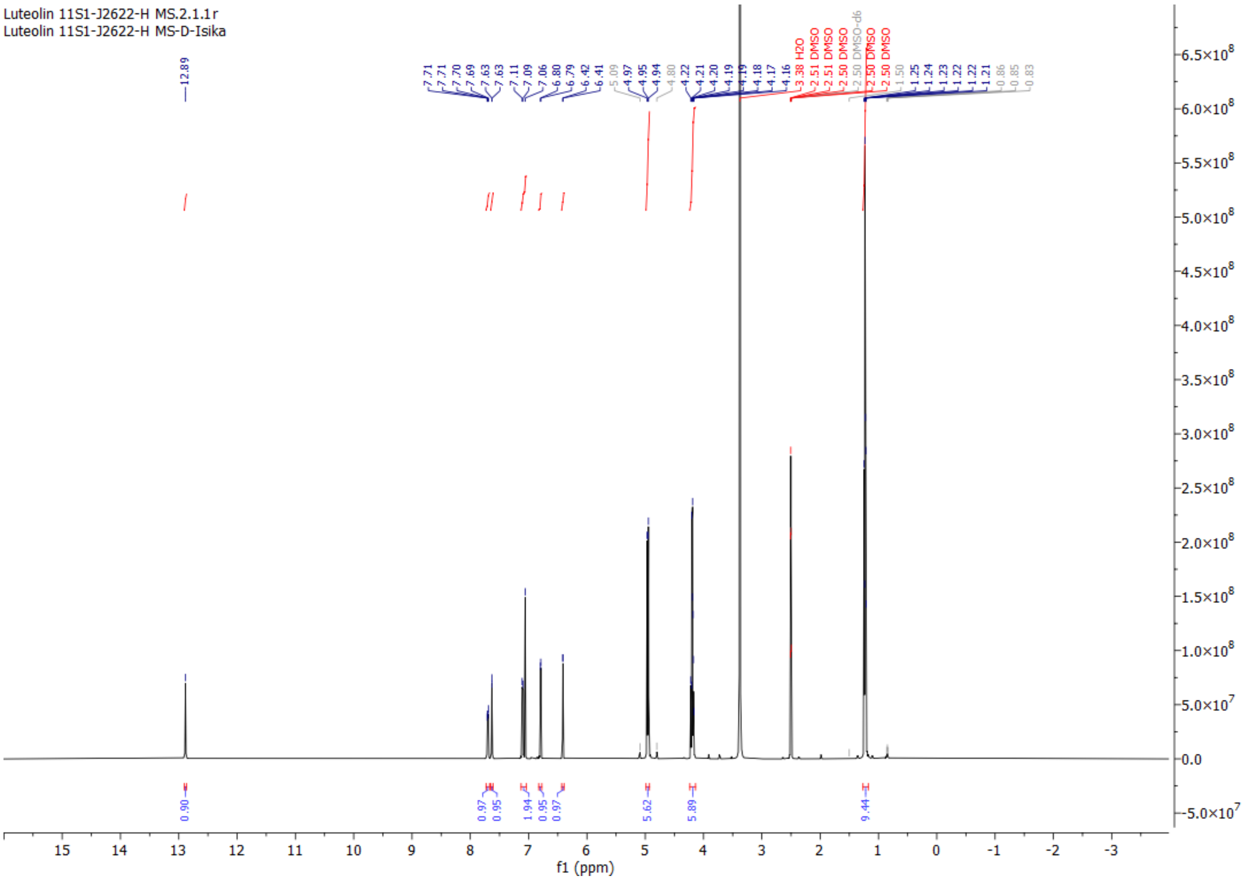

Figure S3: ^1H NMR spectrum (500 MHz, DMSO-d_6): compound 13

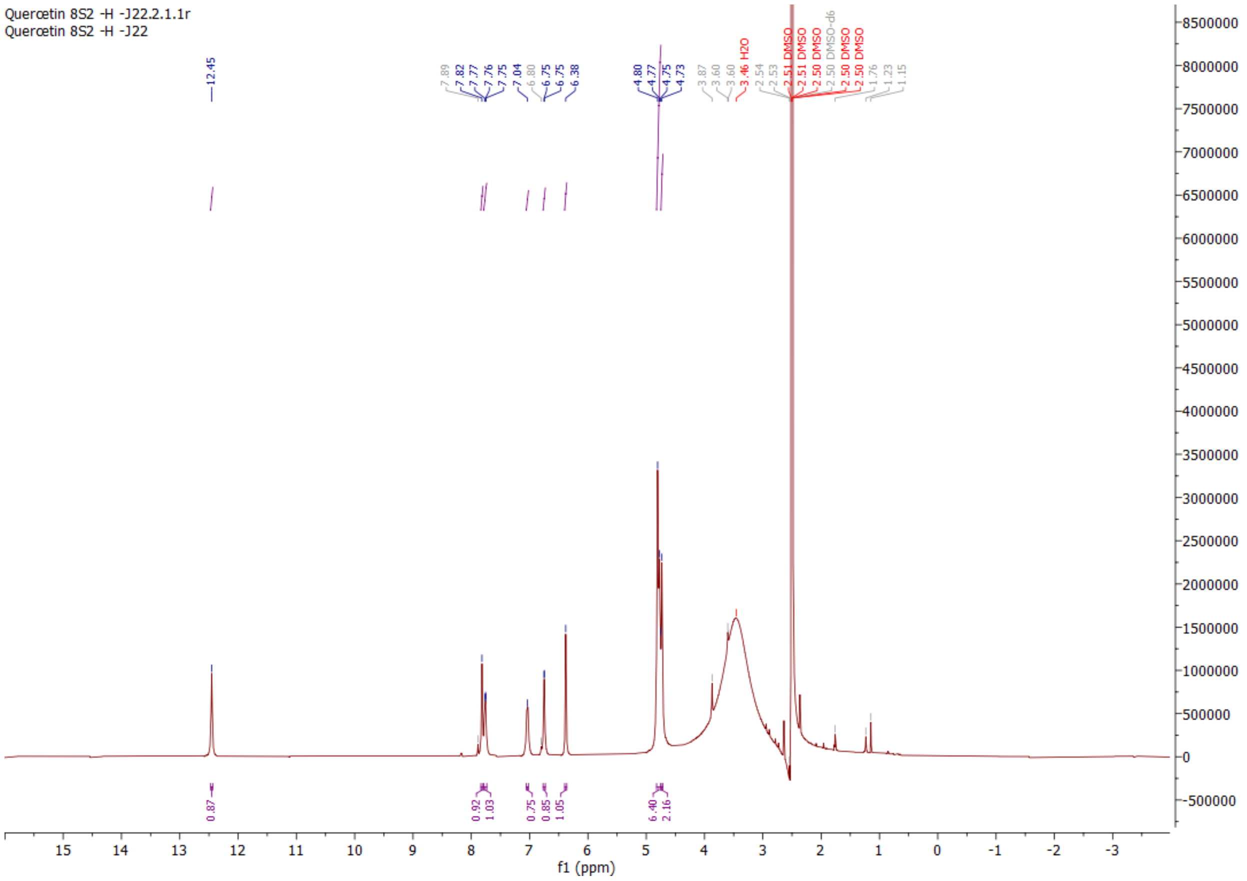

Figure S4: ^{1}H NMR spectrum (500 MHz, DMSO-d$_6$): compound 3

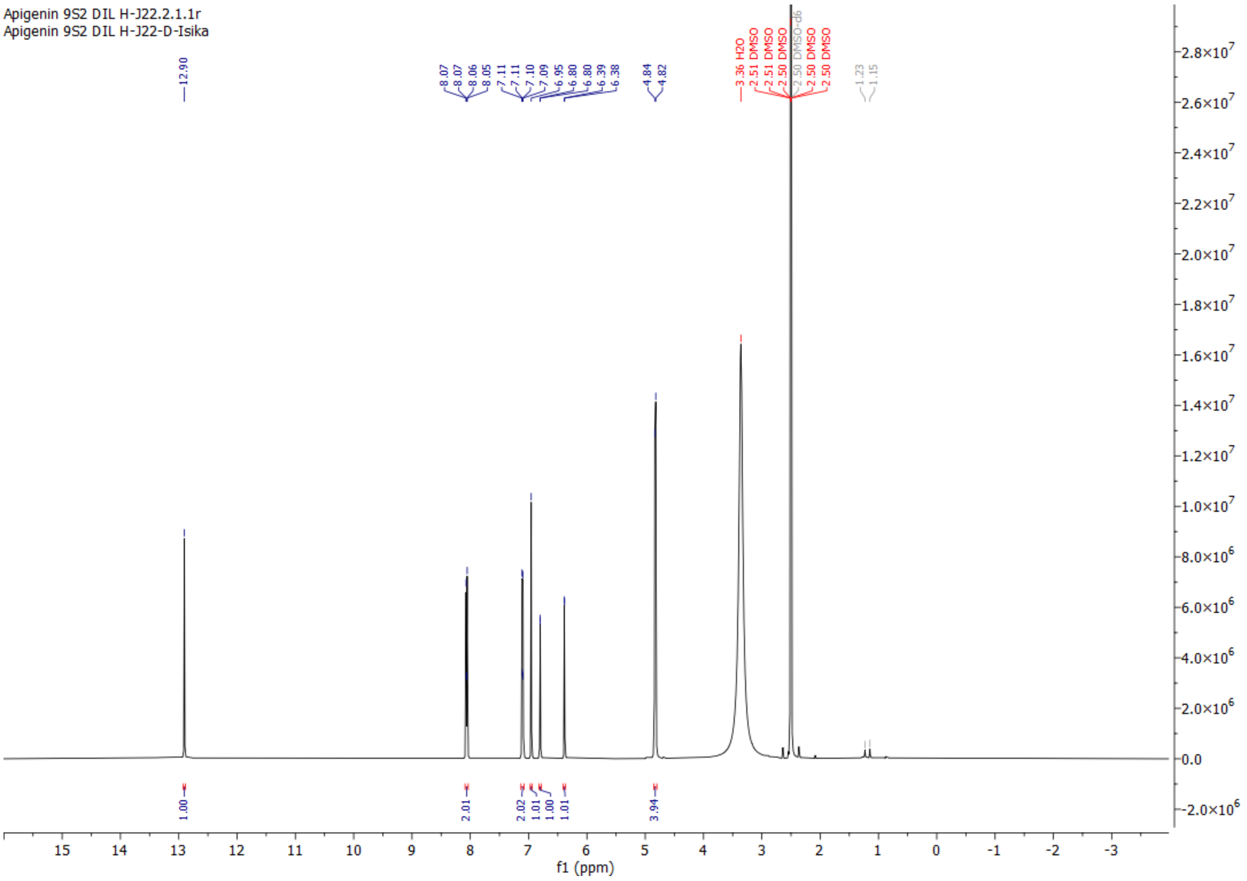

Figure S5: ^1H NMR spectrum (500 MHz, DMSO-d_6): compound 10

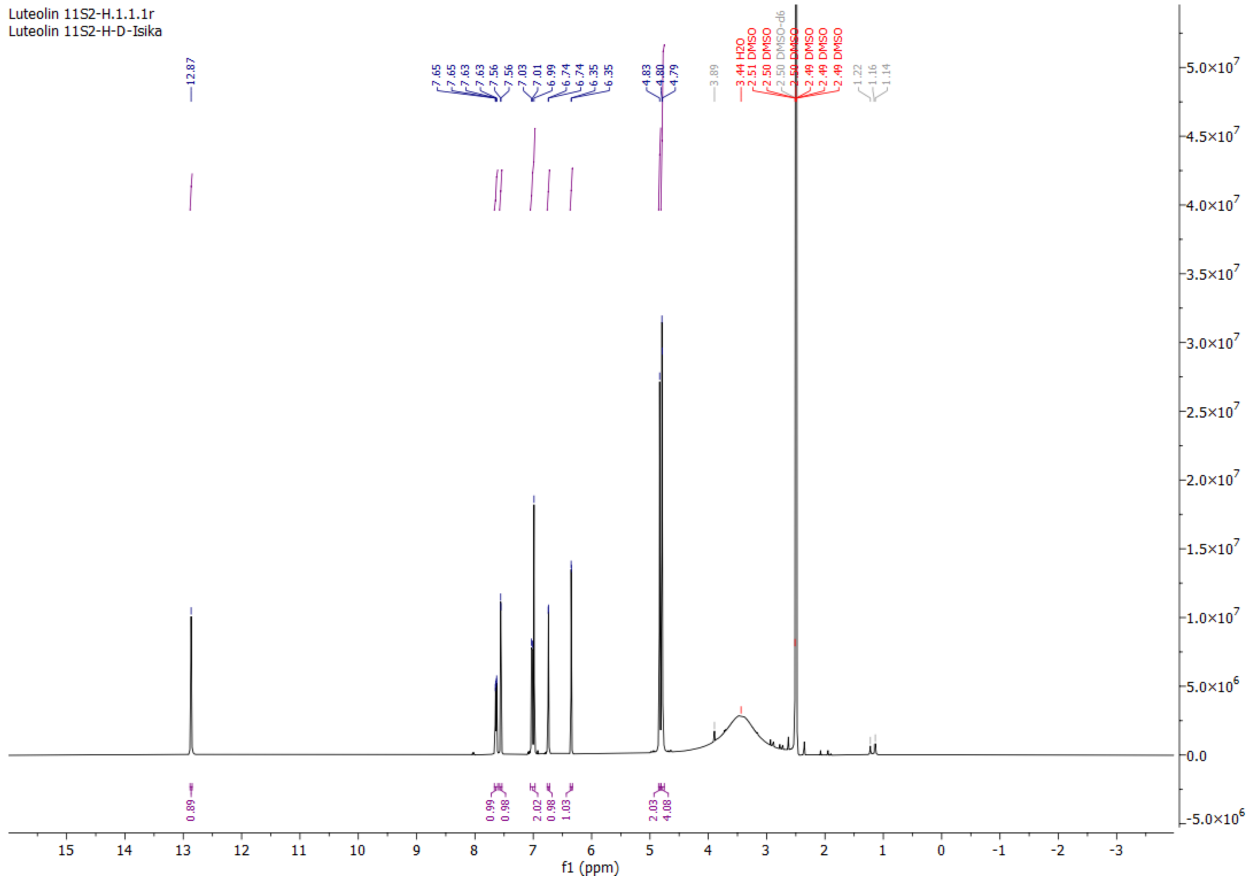

Figure S6: ^{1}H NMR spectrum (500 MHz, DMSO-d_6): compound 14

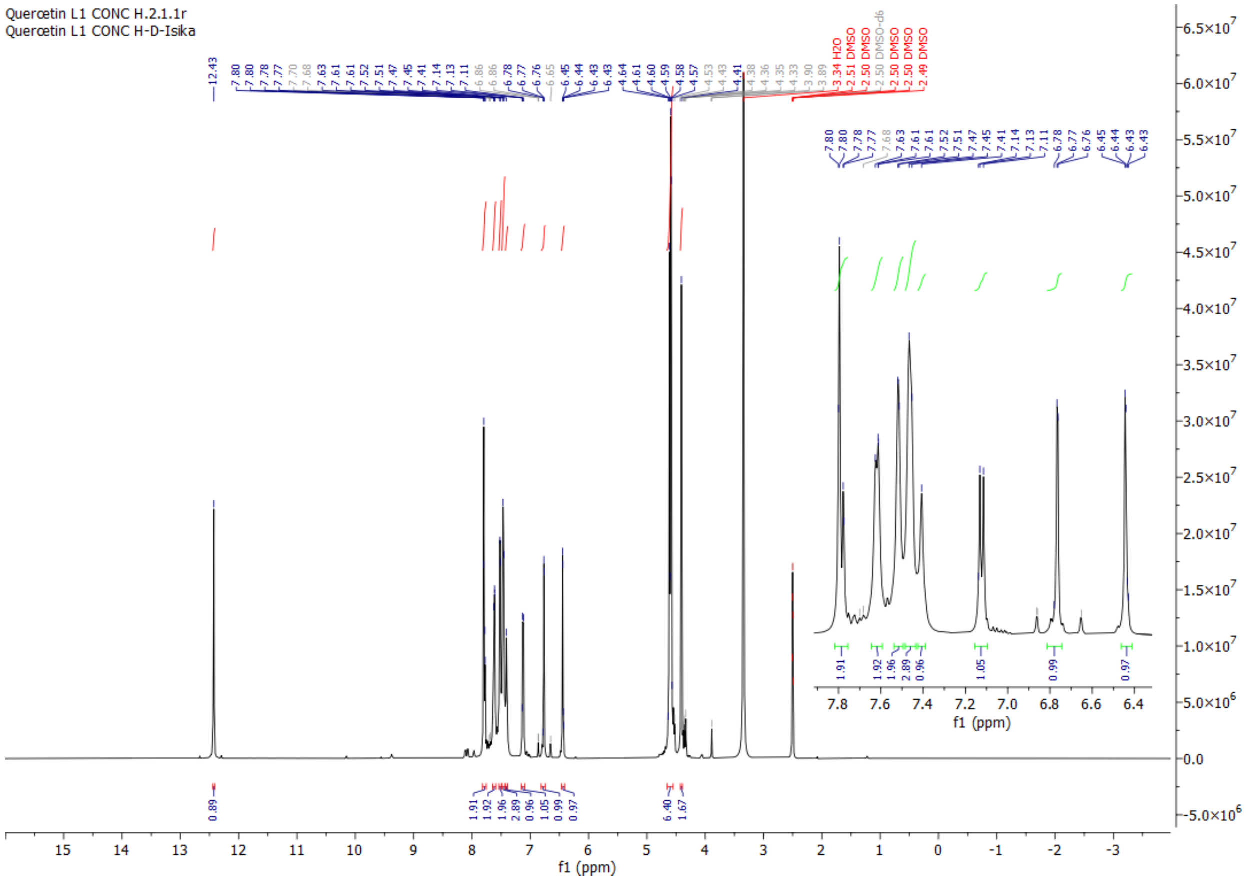

Figure S7: ^1H NMR spectrum (500 MHz, DMSO-d_6): compound 4

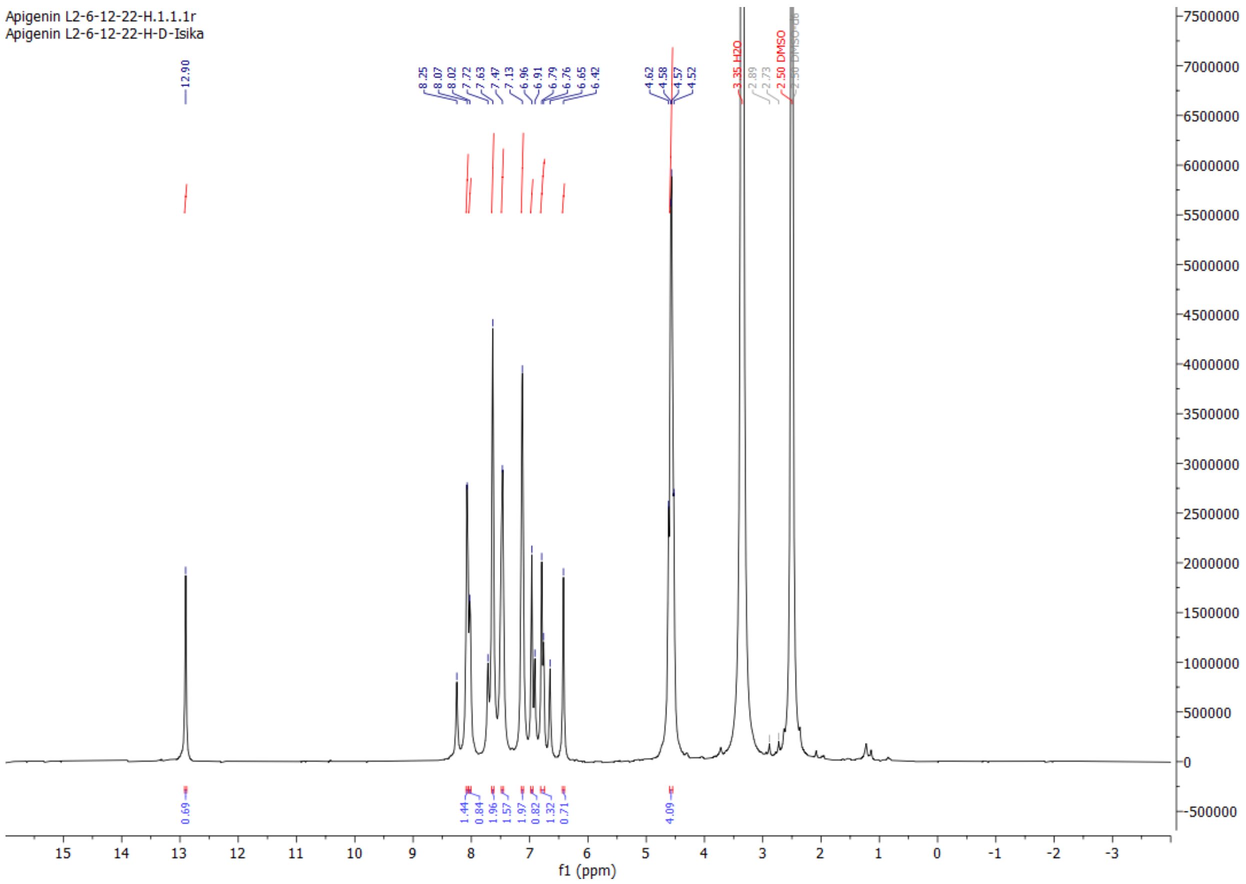

Figure S8: ^{1}H NMR spectrum (500 MHz, DMSO-d_6): compound 11

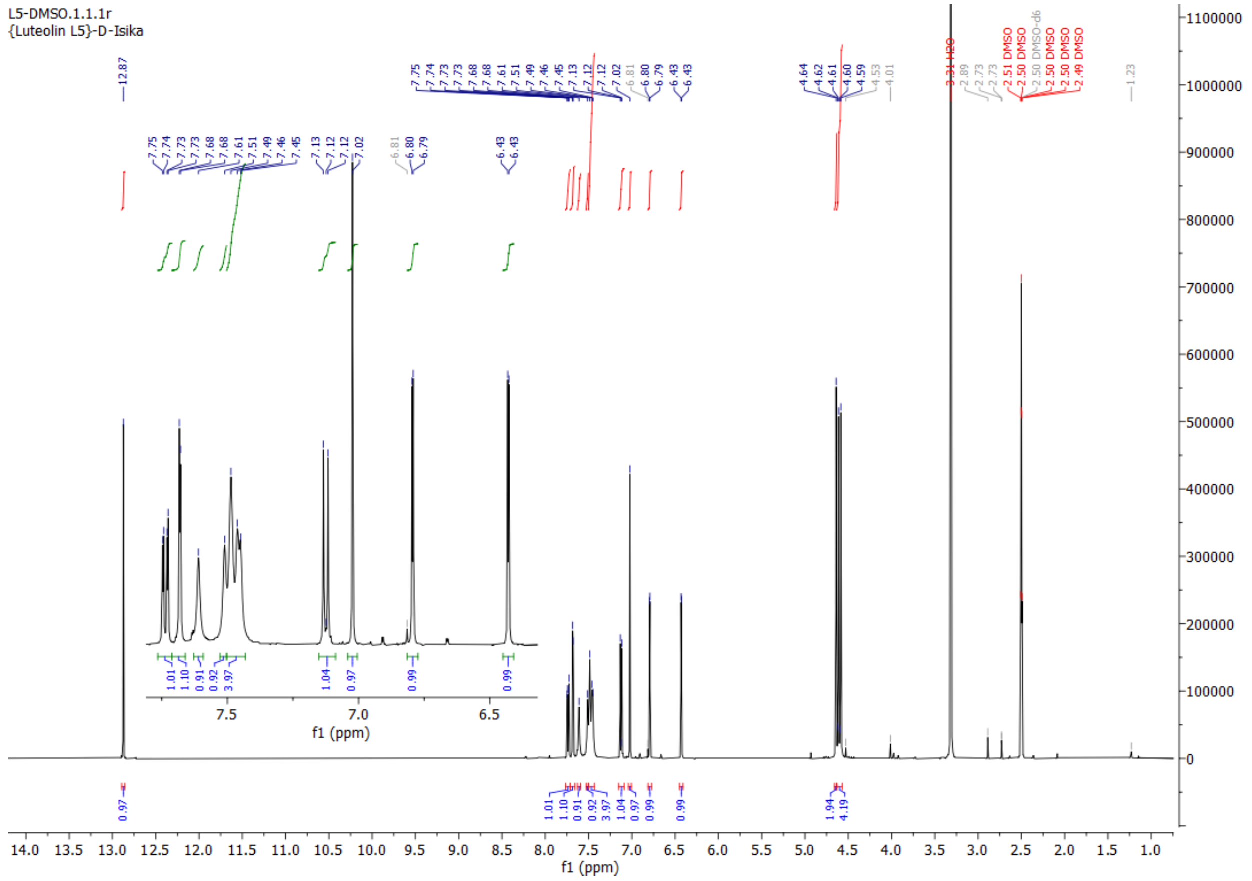

Figure S9: ^1H NMR spectrum (500 MHz, DMSO-d_6): compound 15

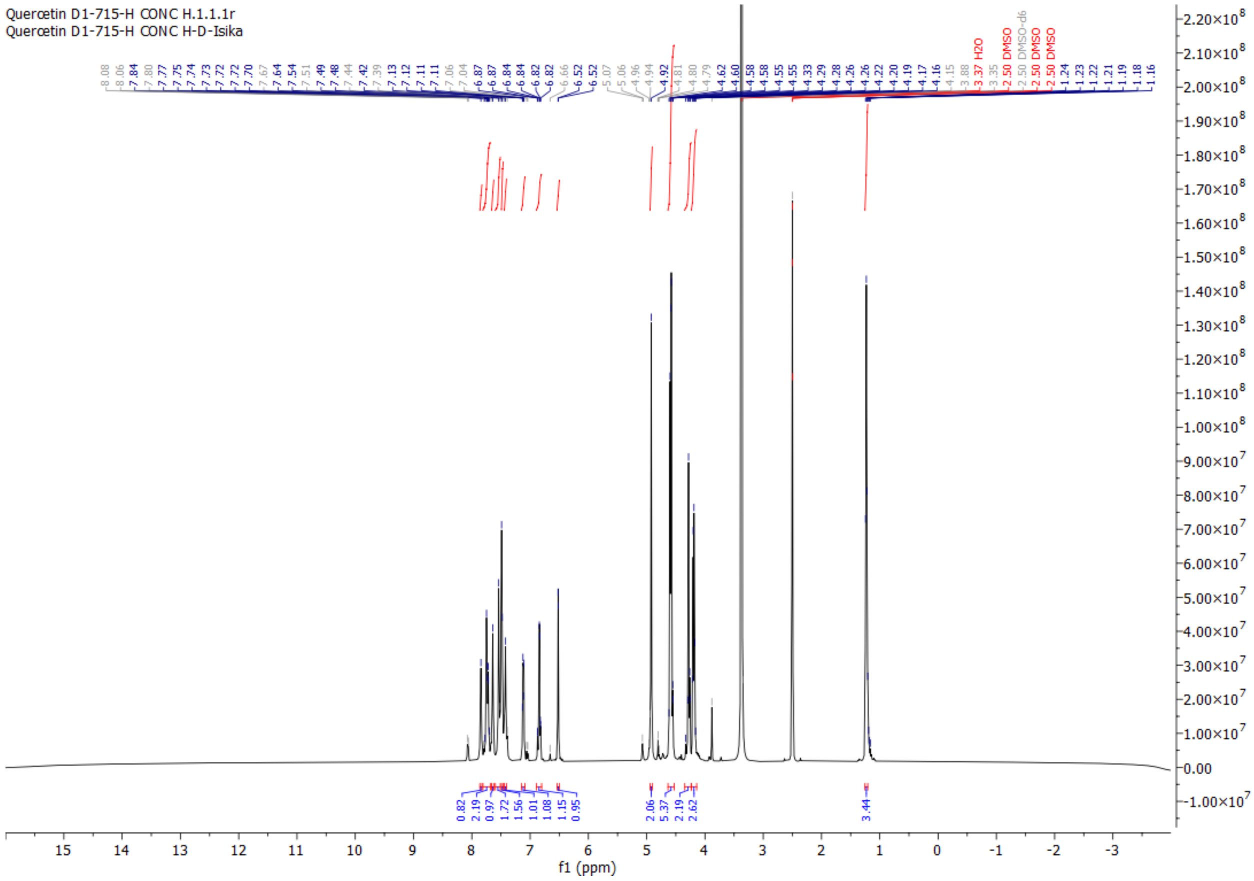

Figure S10: ^{1}H NMR spectrum (500 MHz, DMSO-d6): compound 5

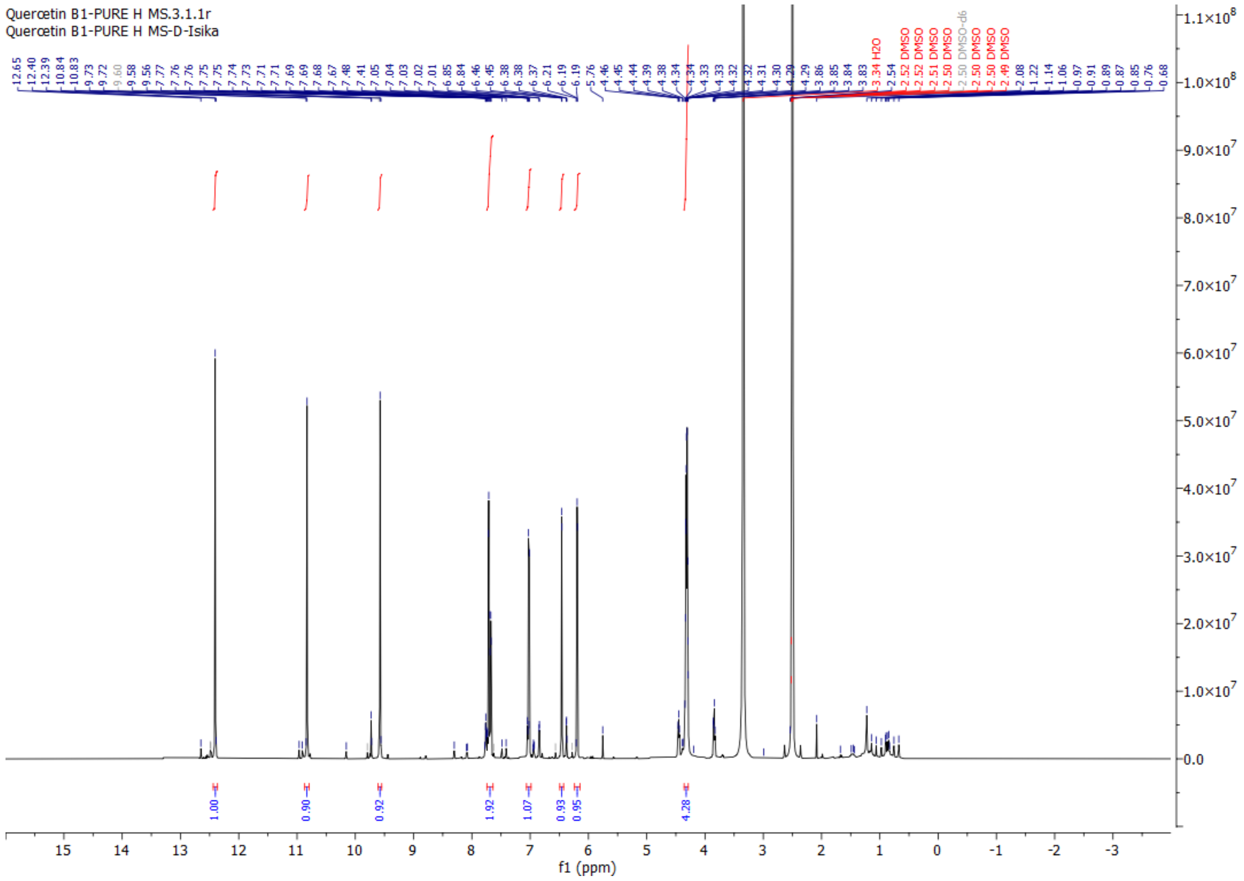

Figure S11: ^{1}H NMR spectrum (500 MHz, DMSO-d_6): compound 6

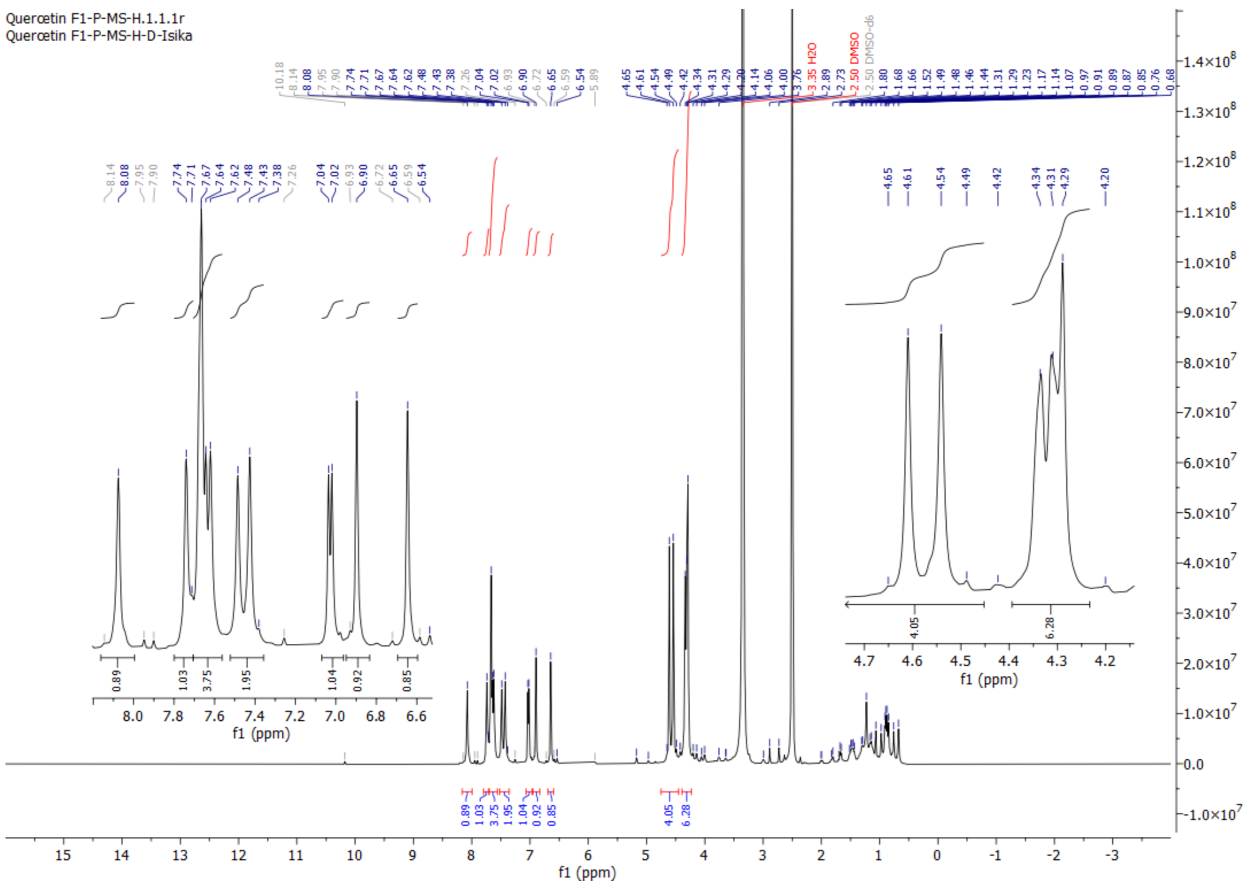

Figure S12: ^{1}H NMR spectrum (500 MHz, DMSO-d_6): compound 7

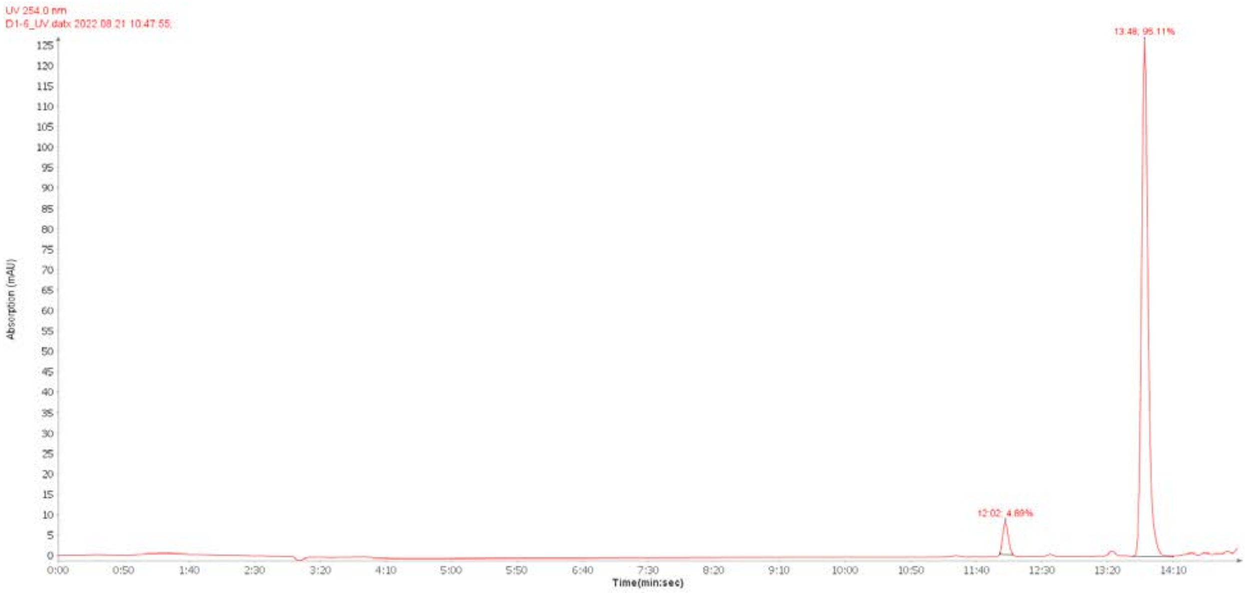

Figure S13: HPLC chromatogram of compound 5

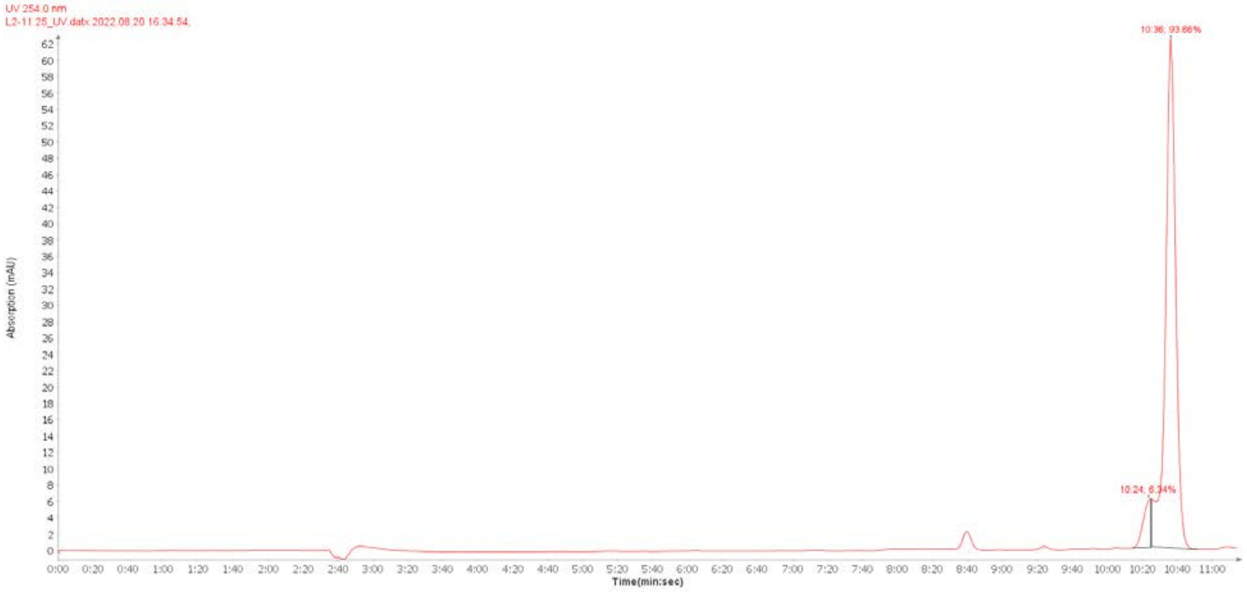

Figure S14: HPLC chromatogram of compound 11

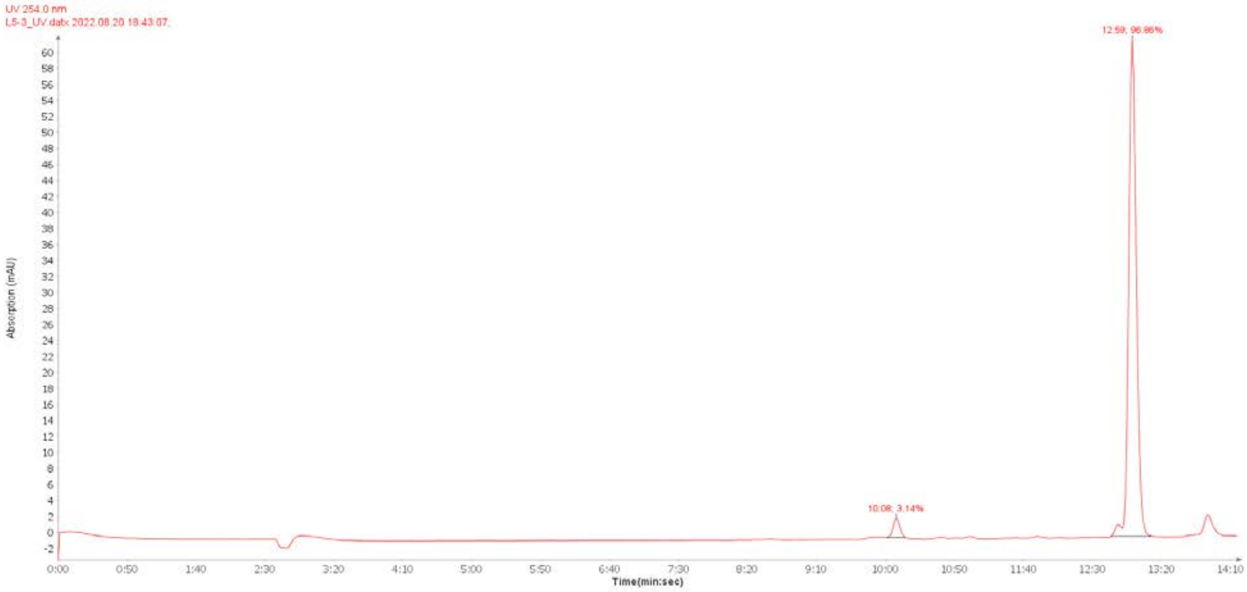

Figure S15: HPLC chromatogram of compound 15

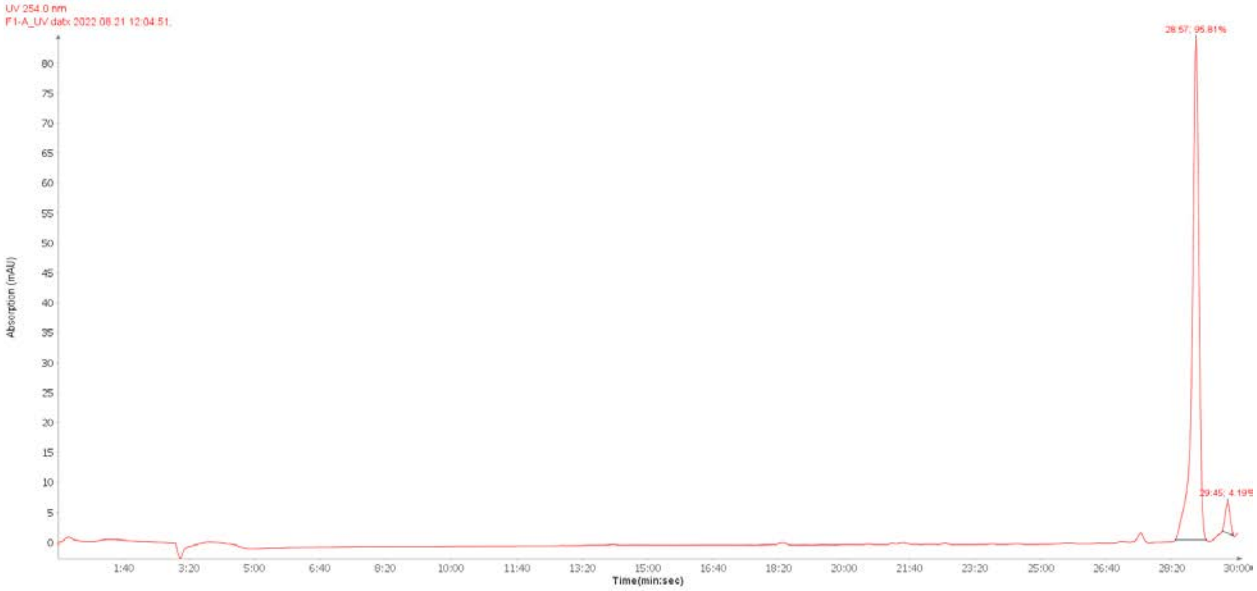

Figure S16: HPLC chromatogram of compound 7

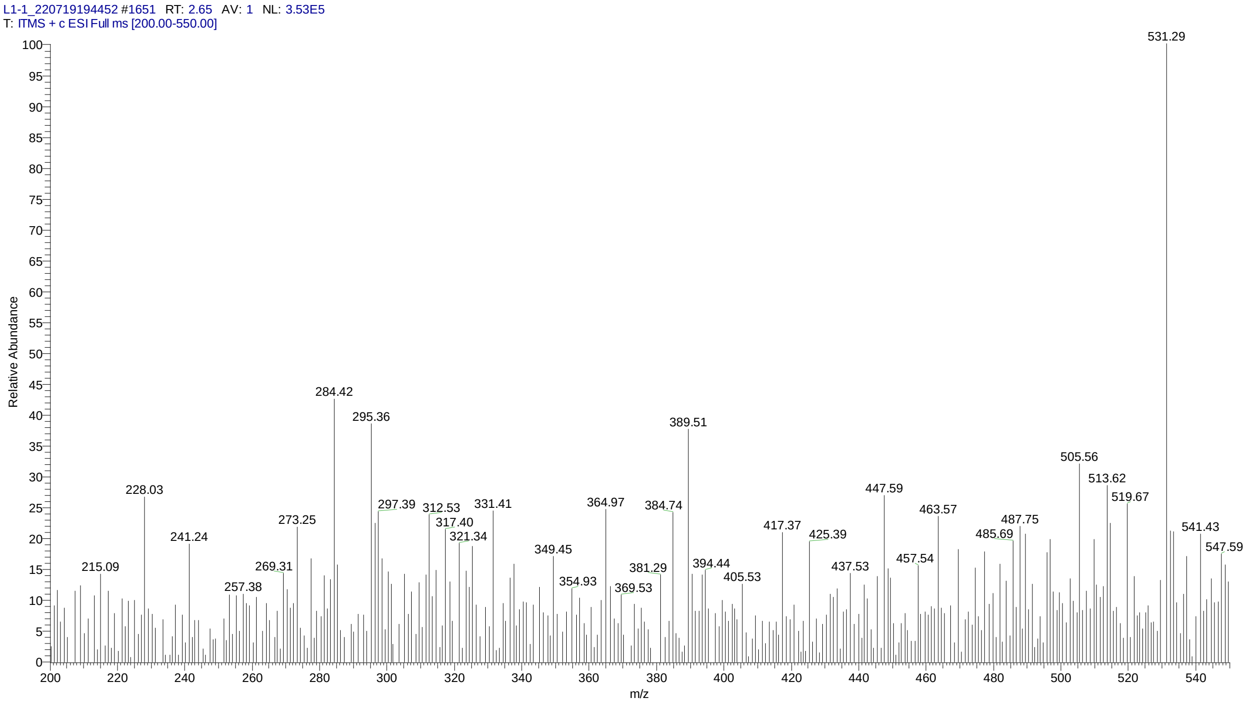

Figure S17: Mass spectrum of compound 4

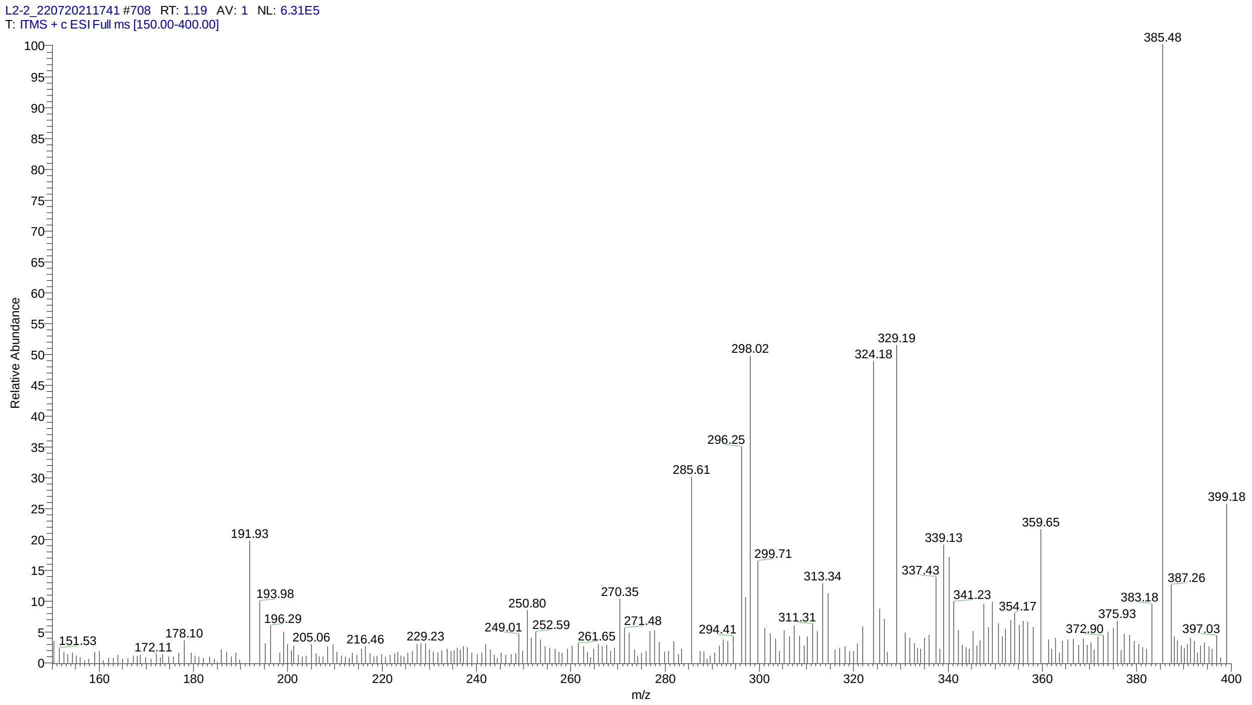

Figure S18: Mass spectrum of compound 11

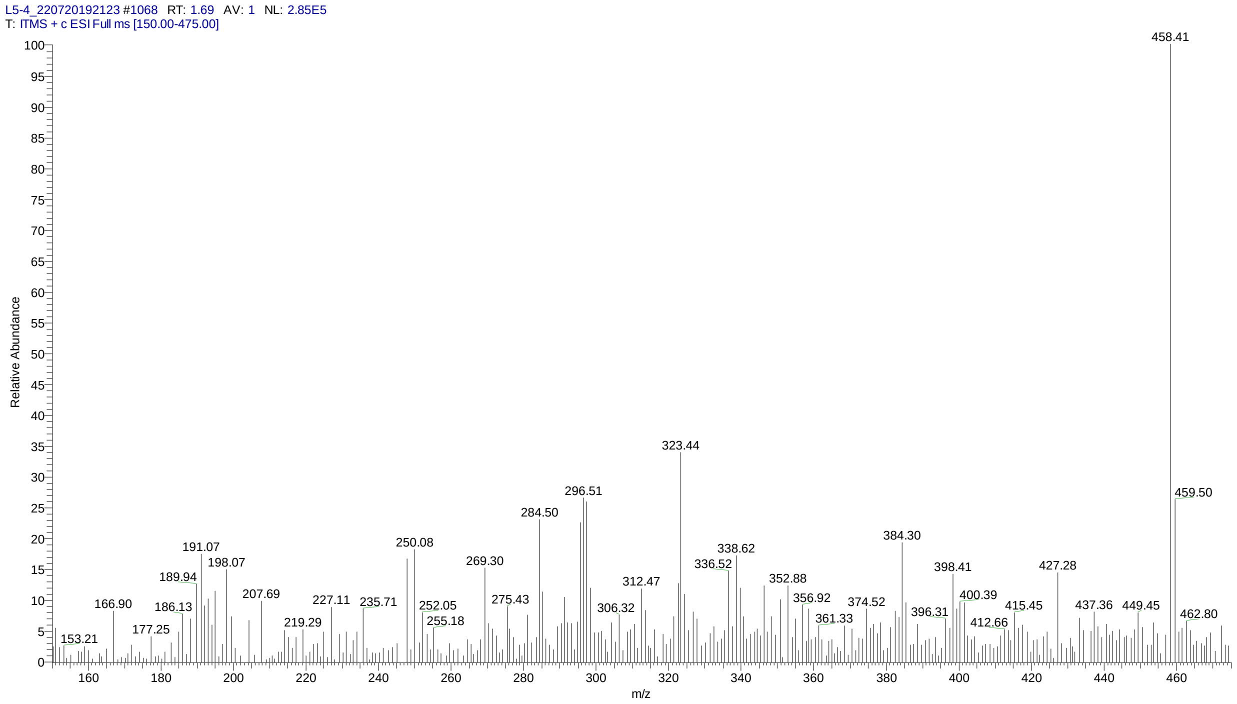

Figure S19: Mass spectrum of compound 15

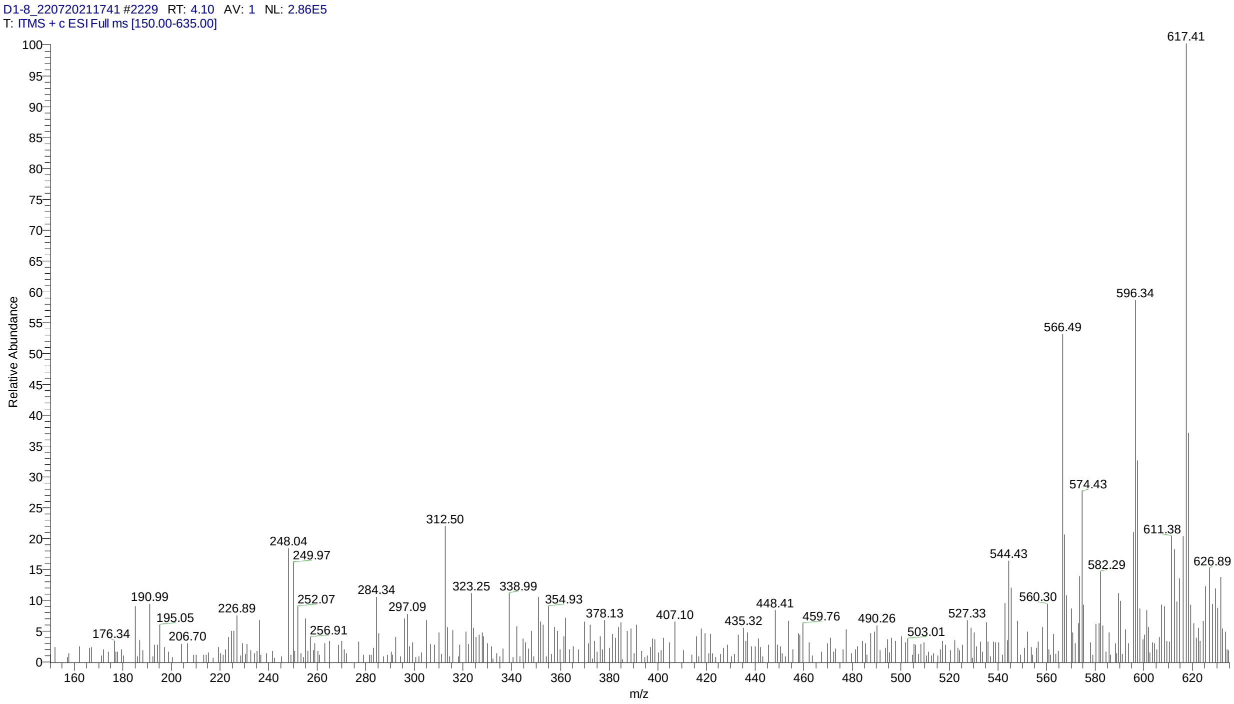

Figure S20: Mass spectrum of compound 5

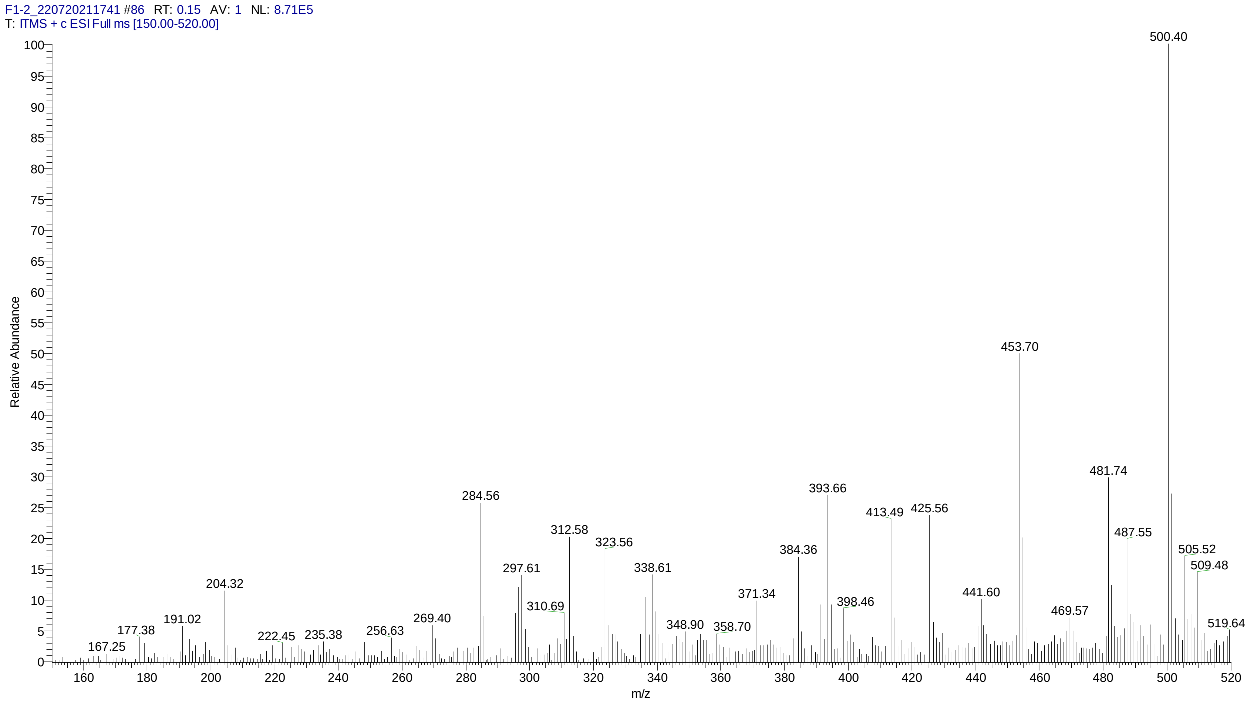

Figure S21: Mass spectrum of compound 7

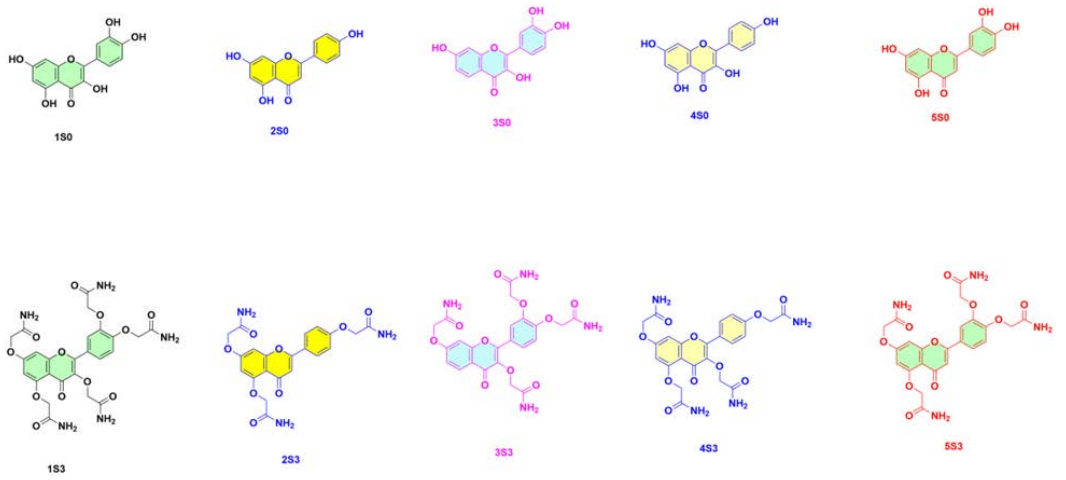

Figure S22: Structure of compounds 1S0-5S0 & 1S3-5S3.
